# CRISPR-Based Genetic Manipulation of *Candida* Species: Historical Perspectives and Current Approaches

**DOI:** 10.3389/fgeed.2020.606281

**Published:** 2021-01-08

**Authors:** Deeva Uthayakumar, Jehoshua Sharma, Lauren Wensing, Rebecca S. Shapiro

**Affiliations:** Department of Molecular and Cellular Biology, University of Guelph, Guelph, ON, Canada

**Keywords:** CRISPR, *Candida*, fungal genetics, functional genomics, gene editing, pathogenesis, drug discovery, biotechnology

## Abstract

The *Candida* genus encompasses a diverse group of ascomycete fungi that have captured the attention of the scientific community, due to both their role in pathogenesis and emerging applications in biotechnology; the development of gene editing tools such as CRISPR, to analyze fungal genetics and perform functional genomic studies in these organisms, is essential to fully understand and exploit this genus, to further advance antifungal drug discovery and industrial value. However, genetic manipulation of *Candida* species has been met with several distinctive barriers to progress, such as unconventional codon usage in some species, as well as the absence of a complete sexual cycle in its diploid members. Despite these challenges, the last few decades have witnessed an expansion of the *Candida* genetic toolbox, allowing for diverse genome editing applications that range from introducing a single point mutation to generating large-scale mutant libraries for functional genomic studies. Clustered regularly interspaced short palindromic repeats (CRISPR)-Cas9 technology is among the most recent of these advancements, bringing unparalleled versatility and precision to genetic manipulation of *Candida* species. Since its initial applications in *Candida albicans*, CRISPR-Cas9 platforms are rapidly evolving to permit efficient gene editing in other members of the genus. The technology has proven useful in elucidating the pathogenesis and host-pathogen interactions of medically relevant *Candida* species, and has led to novel insights on antifungal drug susceptibility and resistance, as well as innovative treatment strategies. CRISPR-Cas9 tools have also been exploited to uncover potential applications of *Candida* species in industrial contexts. This review is intended to provide a historical overview of genetic approaches used to study the *Candida* genus and to discuss the state of the art of CRISPR-based genetic manipulation of *Candida* species, highlighting its contributions to deciphering the biology of this genus, as well as providing perspectives for the future of *Candida* genetics.

## Introduction

Among fungal species, the *Candida* genus has generated interest both as a threat to human health and as an asset to industrial manufacturing. *Candida* pathogens cause a range of infections from common superficial mucosal infections to life-threatening invasive infections, especially amongst immunocompromised individuals. Amongst these species, *Candida albicans* remains one of the greatest threats to human health, with invasive *C. albicans* infection mortality rates as high as 60–72% (Lamoth et al., 2018; Benedict et al., 2019). While *C. albicans* is the most commonly isolated *Candida* pathogen in the clinic, other non-*albicans Candida* species, such as *Candida glabrata*, are rapidly increasing in prevalence, and newly discovered *Candida* species are emerging, such as the multi-drug resistant pathogen *Candida auris* (Geddes-McAlister and Shapiro, 2019). High mortality rates associated with *Candida* infections, coupled with limited antifungal agents, and the emergence of novel drug resistant *Candida* pathogens, calls for a deeper understanding of *Candida* pathogenesis and drug resistance mechanisms (Sharma et al., 2019).

Although *Candida* species are frequently regarded as pathogens, several species possess unique biological processes with prospective commercial benefits. *Candida* species play a role in bioremediation of wastewater and oil spills through the biodegradation of hydrocarbons such as petroleum (Gargouri et al., 2015). One such species is *Candida tropicalis* which is able to use hydrocarbons as their sole carbon source, which allows it to be used in bioremediation processes to degrade harmful substances (Gargouri et al., 2015). Several by-products produced by these species are important to the food processing industry, pharmaceutical industry, and cosmetic industry (Kieliszek et al., 2017). Notably, *Candida krusei* is used extensively in chocolate production, while species such as *C. tropicalis* and *Candida oleophila* produce the extracellular by-product citric acid, which is an important organic acid used in the food industry (Anastassiadis et al., 2005; Max et al., 2010; Dhillon et al., 2011).

Although these *Candida* species can impact both the health and industrial sectors, much is still unknown about their biology, due to limitations in molecular genetic techniques. However, over the last few decades, significant progress has been made toward improving functional genetic techniques as a means to study *Candida* biology. These technologies broadly rely on our ability to targetedly delete, mutate, or otherwise alter a genetic locus in order to study its function via reverse genetics. In this review, we highlight how genetic manipulation techniques have been applied for the study of diverse *Candida* species. We discuss early genetic studies that have made strides to characterize gene function amongst *Candida* species, and focus on CRISPR (Clustered regularly interspaced short palindromic repeats) technology which has contributed significantly to genetic analysis and functional genomics in these critical species. CRISPR has been successfully applied in several *Candida* species, leading to novel insights on aspects of their biology, pathogenesis, antifungal drug resistance, and metabolism. This review describes the current state of the art for CRISPR-based genetic manipulation of *Candida* species, with a focus on how this technology is being applied to further our understanding of the genetics of these complex fungal organisms.

## Genetic Manipulation of *Candida* Species: Historical Context

Despite the relevance of many *Candida* species in both medical and industrial contexts, these organisms remain understudied compared to many other microbial genera of interest. Reverse genetics is a powerful approach used to uncover the function of uncharacterized genes, and thus identify critical genetic factors in fungal biology. In this section, we overview the history of genetic manipulation in *Candida* species, with an emphasis on *C. albicans* as the best-studied *Candida* species with regards to genetic interrogation and the development of tools for functional genetic analysis.

While leveraging genetic approaches to study *Candida* species is important, several unique characteristics in these organisms have hindered progress in this field. A mutation in the leucine tRNA of nine *Candida* species has resulted in their classification as a specific clade (“CTG clad”), based on a resulting alternative codon usage (Gabaldón et al., 2016). These CTG calde *Candida* species, including numerous prevalent pathogens, make use of a unique translational coding system for the CUG codon that encodes serine instead of the universal leucine (Ohama et al., 1993). This alternative codon usage presents a barrier to efficient genetic manipulation, as molecular genetic tools from model organisms, such as *Saccharomyces cerevisiae*, cannot be readily translated for applications in these organisms. Another limitation to genetic analysis in *Candida* organisms is the lack of a complete mating cycle. For the model yeast *S. cerevisiae*, exploiting its simple and tractable mating program has been instrumental for genetic analysis by facilitating the rapid generation of deletion mutants. While mating has been found to occur in some *Candida* species, such as *C. albicans* (Johnson, 2003; Bennett and Johnson, 2005; Hickman et al., 2013), many still lack the ability to mate and the ability to complete the sexual cycle through meiosis. Further, several *Candida* species, including *C. albicans*, are most commonly found as diploid organisms. Together, this has prevented the application of *S. cerevisiae*-based strategies for rapid genetic manipulation through mating, meiosis, and selection.

**Figure 1:**
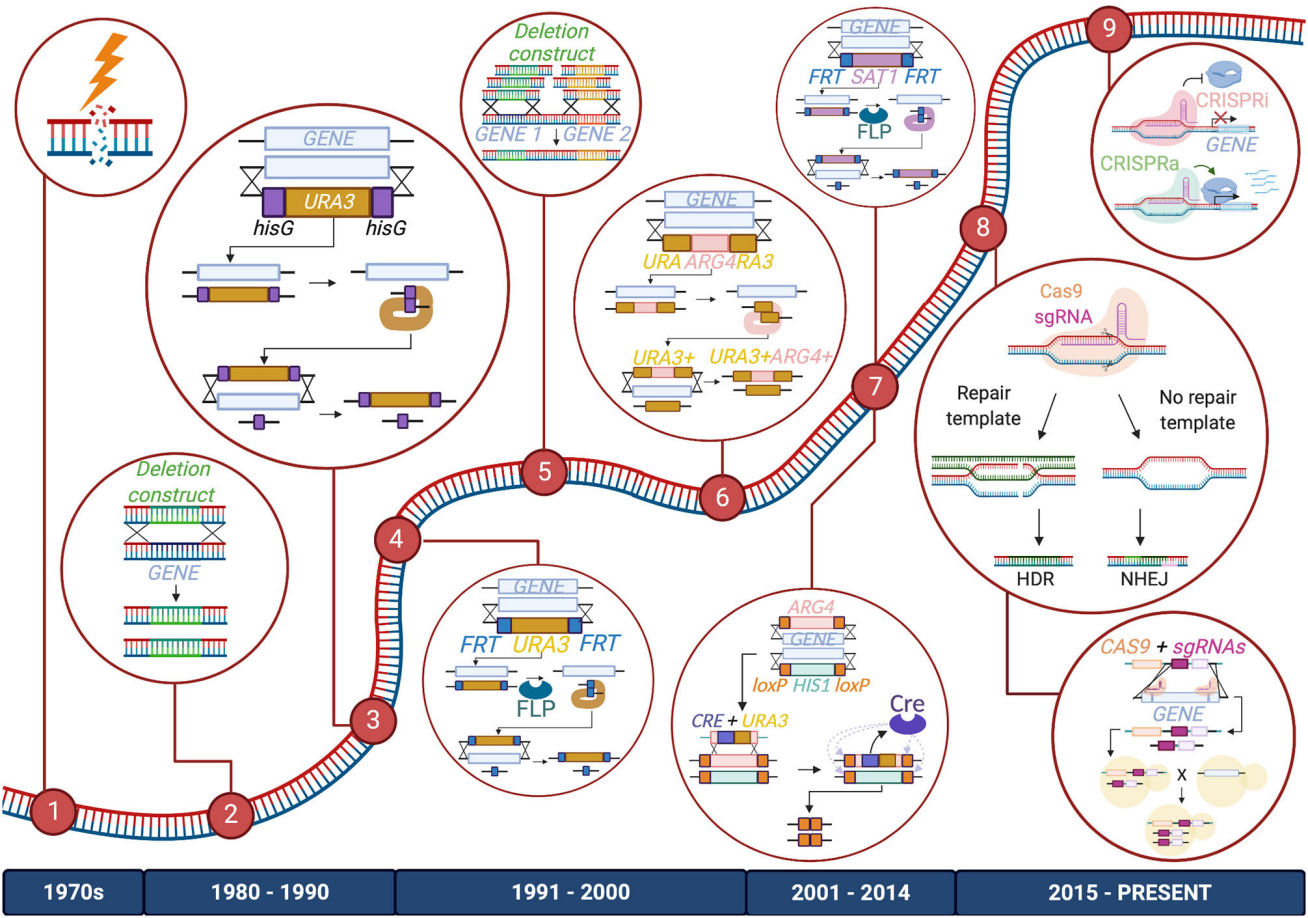
Timeline of advancements made to *C. albicans* gene manipulation technology. Major methods included in the *C. albicans* gene editing toolbox are listed from left to right: (1) UV exposure and chemical mutagenesis; (2) Homology-directed repair (HDR) via transformation of a deletion construct or a repair template; (3) URA Blaster; (4) URA Flipper; (5) HDR via transformation of PCR-amplified deletion constructs or repair templates; (6) UAU1 cassette; (7) Cre-LoxP and SAT1/caNAT1 Flipper; (8) CRISPR-Cas9 and CRISPR-Cas9 gene drives; (9) CRISPR interference (CRISPRi) and CRISPR activation (CRISPRa). The diagram depicts these tools as they are used to create homozygous gene deletions, except for CRISPRi and CRISPRa, which are depicted to repress or activate genes, respectively. Enzymes, DNA, and RNA components are not drawn to scale.

The genetic toolbox used to study *Candida* species began to take shape in the late 20th century, as researchers developed creative methods to study the *Candida* genus; most of these technological milestones were achieved first in *C. albicans* and then adapted for use in other species. Early studies investigating the *Candida* genome relied on UV exposure or chemical mutagenesis to randomly induce mutations in these organisms. However, more targeted and less toxic techniques have since been applied (Kelly et al., 1987; Fonzi and Irwin, 1993; Figure 1).

One of the earliest systems for targeted genetic manipulation in *C. albicans* was the URA Blaster (Alani et al., 1987; Fonzi and Irwin, 1993; Lay et al., 1998), which is comprised of the *C. albicans URA3* gene flanked by *hisG* sequences from *Salmonella enterica* Typhimurium, and homology regions of the target gene. This cassette can be transformed into a uridine auxotrophic strain and integrated into the target locus. Because two *hisG* sequences in close proximity are potentially unstable, they can spontaneously undergo recombination to excise the *URA3* sequence, allowing for a sequential transformation that targets the second allele. This clever system facilitates the homozygous deletion of a gene of interest, which is very powerful for diploid organisms such as *C. albicans*. While this method was an instrumental part of the development of methods of genetic analysis in *C. albicans*, it also has limitations. Firstly, the excision of the marker occurs at a rare frequency of 0.01%. When it is successful, the construct still leaves behind a sizable *hisG* sequence at the target site, which could impact adjacent gene expression or be involved in further gene rearrangement events. Recombination between *hisG* sequences can also be imprecise, leading to the deletion of neighboring sequences (García et al., 2001). Choosing to express *URA3* at an ectopic site can also reduce the virulence of the strain and result in a phenotype that is mistakenly associated with the mutation of the gene at the target locus (Alani et al., 1987; Lay et al., 1998; Cheng et al., 2003; Brand et al., 2004; Samaranayake and Hanes, 2011). To avoid misleading impacts on virulence, the *URA3* gene can be re-inserted at highly expressed loci or its native locus to reconstitute the wild-type *URA3* phenotype (Davis et al., 2000; Murad et al., 2000; Sundstrom et al., 2002; Ramón and Fonzi, 2003; Brand et al., 2004).

*URA3* marker recycling and subsequent reconstitution were made easier with an improved version of the URA Blaster, dubbed the URA Flipper, which was developed to address some of the shortcomings of its predecessor (Morschhauser et al., 1999). In this construct, the *URA3* gene is fused with a codon-optimized FLP recombinase gene, under the control of a *SAP2* inducible promoter, and flanked by FLP recognition target (FRT) sequences and homology regions of the target gene. Unlike the URA Blaster, once the cassette inserts itself into the target locus, it can be excised efficiently by inducing expression of FLP recombinase to catalyze the recombination of FRT sequences. This process excises the construct, leaving only a short FRT scar at the target locus after marker recycling.

The URA Blaster and URA Flipper systems rely on the use of auxotrophic strains and also require additional transformation steps to target both alleles of a gene. Given the additional steps needed to remove *URA3* from the ectopic site, a number of modifications were made to streamline this approach. The use of triple auxotrophic strains, such as BWP17 (*ura3*Δ, *his1*Δ, and *arg4*Δ), enabled the ability to incorporate two different auxotrophic selection markers and target two alleles (Wilson et al., 1999; Enloe et al., 2000; Noble et al., 2010). Yet another recombinase-based gene-editing tool, the Cre-loxP system, was designed to take advantage of these triple auxotrophic strains. Two selection markers, each flanked by loxP sites and homology regions to the gene of interest, are used to target both alleles of a gene simultaneously (Dennison et al., 2005). A third cassette, containing a Cre recombinase gene under the control of an inducible *MET3* promoter, is fused to a *URA3* marker designed to replace one of the selection markers, and insert itself in between two loxP sites. Cre recombinase can be expressed to catalyze its own removal via recombination of the loxP sites, as well as that of the other cassette at the other allele. Although efficient at marker recycling, the Cre-LoxP system still requires multiple transformation steps and the use of three selection markers, preventing it from being applicable to all *C. albicans* strains (Dennison et al., 2005; Papon et al., 2012).

A modified version of the URA Blaster approach, the UAU1 cassette, was developed, where the *ARG4* marker is placed in the middle of the 5′ and 3′ ends of a *URA3* gene that share homology with each other, which in turn are surrounded by homology regions to the target gene (Enloe et al., 2000). Once the cassette inserts into the target locus via homologous recombination, the *ARG4* marker can be excised to form a complete *URA3* gene. Using this clever approach enables researchers to streamline the generation of homozygous mutants, as selection of both *URA3* and *ARG4* markers indicates that both alleles of the target gene have been replaced (Enloe et al., 2000). To perform large-scale mutagenesis of the genome, the UAU1 cassette was inserted into a Tn7 bacterial transposon plasmid, which randomly inserts the cassette into the *C. albicans* genome for subsequent selection of successful deletion strains (Davis et al., 2002; Xu et al., 2011). While the UAU1 cassette does not allow marker recycling and relies on the use of strains with multiple auxotrophies, this innovative system was amongst the first to enable large-scale gene disruption in *C. albicans*, and was used to identify putative essential genes based on failure to obtain deletion mutants of certain loci (Enloe et al., 2000).

To circumvent any undesirable outcomes from the use of auxotrophic strains, and to enable widespread genetic modifications in strains lacking specific auxotrophies, such as clinical isolates, drug selection markers were developed for use in *Candida* species. The *URA3* marker in the URA Flipper was first replaced with a mycophenolic acid resistance marker, *IMH3*, to successfully knock out efflux pump genes in both *C. albicans* and *Candida dubliniensis* (Wirsching et al., 2000b, 2001). However, mycophenolic-resistant transformants were slow-growing and the *IMH3* gene used in the selection marker, had a tendency to recombine with the native *IMH3* locus, rather than at the target loci. As a result, *IMH3* was later replaced by genes encoding streptothricin acetyltransferase (*SAT1*) from bacterial transposon Tn1825, or nourseothricin acetyltransferase (*caNAT1*) from *Streptomyces noursei*, both conferring nourseothricin resistance (Wirsching et al., 2000a; Reuss et al., 2004; Shen et al., 2005). The SAT1 Flipper and caNAT1 Flipper, respectively, could rapidly produce nourseothricin-resistant transformants, and were found to negligibly impact cell growth and physiology of resulting mutants (Shen et al., 2005). The nourseothricin resistance markers have been applied for use in various *Candida* clinical isolates, including strains of *C. albicans, Candida parapsilosis, Candida lusitaniae, C. glabrata*, and *Candida kefyr* (Shen et al., 2005).

Despite these tools, the creation of multi-gene deletion strains or large deletion libraries still remained a daunting task, yet many research groups embarked on this challenge. To date, several large-scale *Candida* mutant libraries have been generated for the following: the study of gene function using single- and double- homozygous mutants; the study of essential genes through the production of conditional expression mutants or heterozygous strains utilizing the GRACE method (gene replacement and conditional expression), where one copy of the allele is knocked out and the remaining wild-type allele is placed under an inducible promoter; large-scale functional analysis of genes using transposon mutagenesis platforms; the creation of barcoded gene deletion libraries for subsequent *in vivo* studies; and the fusion of genes to epitope or fluorescent markers to monitor downstream protein localization (Gerami-Nejad et al., 2001; Davis et al., 2002; Roemer et al., 2003; Nobile and Mitchell, 2009; Noble et al., 2010; Segal et al., 2018). These libraries have been used to conduct diverse functional genomic analyses, uncovering new genes involved in all aspects of *Candida* biology, from the fundamentals of pathogenesis to drug target discovery (Noble et al., 2010; Oh et al., 2010; Ryan et al., 2012; Schwarzmüller et al., 2014; O'Meara et al., 2015; Motaung et al., 2017).

## CRISPR Technology to Study *Candida* Species

Although these techniques have provided significant advancements in the study of *Candida* genetics, new tools continue to be developed to improve functional genomic studies. CRISPR is a gene editing tool that has revolutionized the efficiency of genetic analysis in innumerable organisms, including many previously intractable microbial species and several *Candida* species (Shapiro et al., 2018a; Román et al., 2019b; Morio et al., 2020). This genome editing system is a complex that consists of two key molecular components: a CRISPR-associated (Cas) endonuclease enzyme, and a single guide RNA (sgRNA). The sgRNA guides the CRISPR-Cas complex to a precise DNA location where it induces a double-stranded break (DSB) through Cas's endonuclease activity (Dominguez et al., 2015). The sgRNA is itself comprised of two main components: the crRNA, which is a short sequence (the “spacer”) that locates a precise location in the target genome (the “protospacer”) based on complementary base pairing, and the tracrRNA, which binds the Cas protein (Mohanraju et al., 2016). In order for the CRISPR complex to target the correct genomic location, it requires that the target sequence be adjacent to a protospacer adjacent motif (PAM) in the genome (Russa et al., 2015). This short sequence allows the CRISPR complex to differentiate between the target and the sgRNA sequence (Russa et al., 2015). For *Streptococcus pyogenes* Cas9 (*Sp*Cas9), one of the most widely used CRISPR systems, the PAM sequence is NGG (Russa et al., 2015). This can be a limiting factor when designing sgRNAs, as the PAM sequence is not uniformly abundant in all organisms. To circumvent this, other Cas proteins can be used for CRISPR editing, that each possess a unique PAM sequence, and thus a different targeting range.

CRISPR's efficiency as a genome editing system is based on pre-existing cellular mechanisms that repair DNA double-stranded breaks (DSBs). The two most common repair strategies are homology-directed repair (HDR) and non-homologous end-joining (NHEJ) (Sander and Keith Joung, 2014). The first strategy, HDR, repairs DSBs by using DNA that is complementary to the damaged loci as a template (Sander and Keith Joung, 2014). This repair mechanism can be exploited by providing the cell with an exogenous template DNA strand that contains either specific mutations or novel DNA sequences to be incorporated into the targeted locus (Sander and Keith Joung, 2014). The other DSB repair strategy, NHEJ, introduces insertions or deletions (indels) into the DSB region upon re-joining the cleaved DNA (Maruyama et al., 2016). In the past few years, CRISPR technologies have been developed for rapid genome editing in several *Candida* species, as described below.

### CRISPR Technology Development in *Candida albicans*

**Figure 2:**
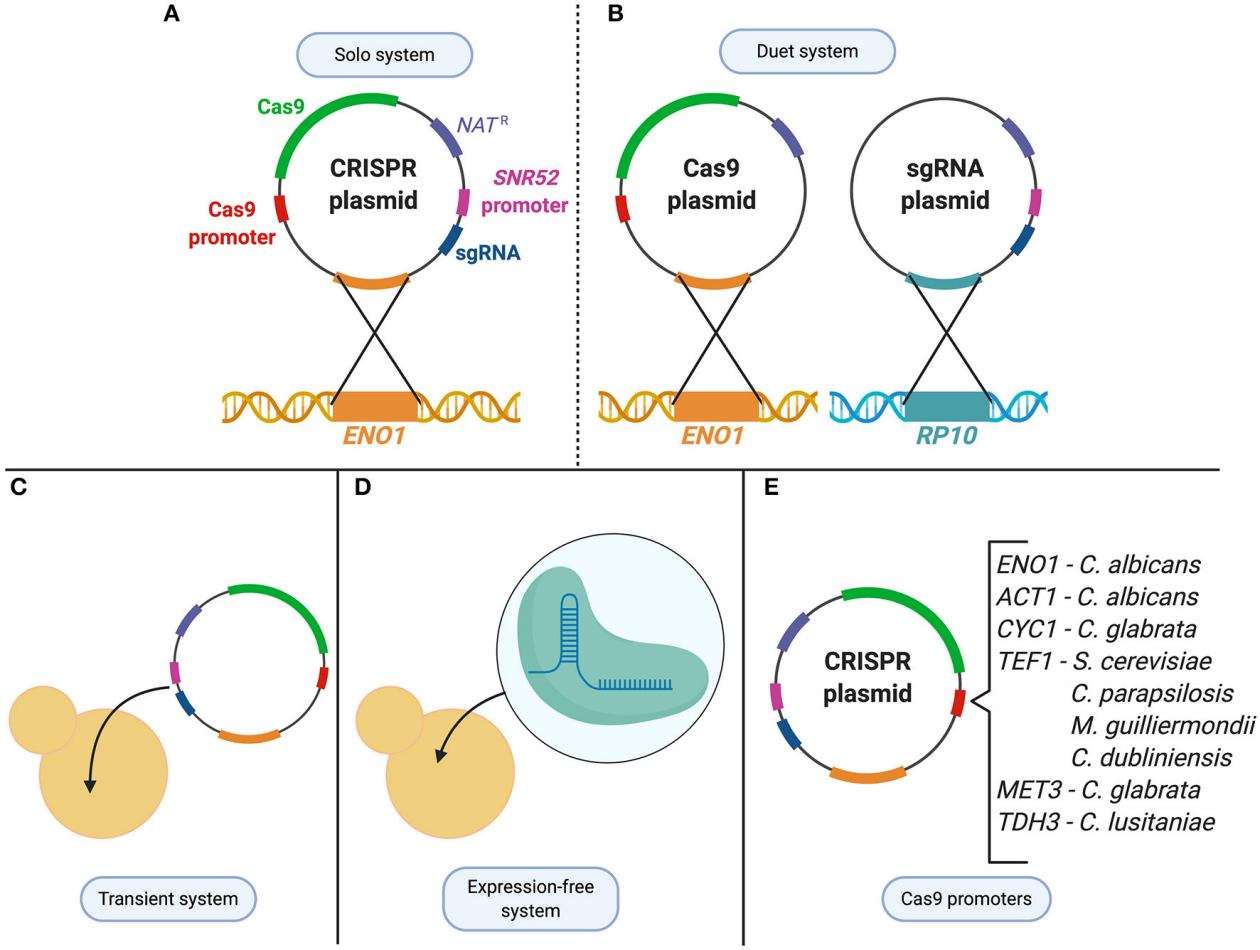
CRISPR technologies used to study *Candida* species. **(A)** *C. albicans* solo system consists of a single CRISPR plasmid that contains both the Cas9 enzyme and the sgRNA which is then integrated at the *ENO1* locus. **(B)** *C. albicans* duet system in which the CRISPR system is divided into two plasmids and integrated at two different loci (*ENO1* and *RP10*). **(C)** Transient system used in *C. albicans, C. lusitaniae, C. glabrata, and C. tropicalis* as an alternative expression method to integration of the CRISPR system. **(D)** To avoid changes to the expression system from species to species, an expression-free system, in which purified Cas9 and CRISPR RNAs are complexed to form ribonucleoproteins (RNPs), can be transformed into the cell. **(E)** Cas9 expression controlled under various promoters depending on the species it is being applied to.

Within the *Candida* genus, CRISPR was first developed in the most widely studied species, *C. albicans*, before being applied to other *Candida* organisms (Figure 2). The first application of CRISPR in *C. albicans* was in 2015 by Vyas et al. who adapted a Cas9 nuclease and sgRNA system from *S. cerevisiae* and optimized it for use in *C. albicans* (Vyas et al., 2015). Due to the alternative CTG usage, the *CAS9* nuclease-encoding gene for *C. albicans* needed to be optimized to avoid misincorporation of serine amino acids into the nuclease. Since *C. albicans* is unable to maintain autonomously-replicating plasmids, the CRISPR plasmid system was designed to integrate into the *C. albicans* genome, and the endogenous RNA polymerase III promoter, *pSNR52*, was used to express the sgRNA (Vyas et al., 2015). Two systems were initially created, a solo and a duet system, in which either one or two plasmids were used to express Cas9 and the sgRNA, respectively (Figure 2). Both systems supplied a repair template using HDR to target a gene by introducing a frameshift mutation that led to a premature stop codon (Vyas et al., 2015). The systems showed a mutation frequency of between 20–40% and 60–80% for the duet and solo system, respectively (Vyas et al., 2015). This constituted the first use of CRISPR in *C. albicans*, and described a system which could mutate genes in both alleles of this diploid, as well as several copies of a multigene family from a single transformation (Vyas et al., 2015). This discovery was a milestone in *C. albicans* research, providing a foundation for continuous advancements in the field.

The CRISPR system described in Vyas et al. involved the stable integration of the plasmid encoding the CRISPR components directly into the genome of *C. albicans*. To avoid concerns of any off-target effects that could result from continuous CRISPR activity, a transient approach was developed to express the system without genome integration (Figure 2). Min et al. created a transient system that expressed Cas9 and sgRNA targeting the *ADE2* reporter gene in *C. albicans* (Vyas et al., 2015; Min et al., 2016). A nourseothricin resistance selection marker was supplied on a repair template that was used to successfully disrupt the *ADE2* gene upon DSB repair. This proved that plasmid integration and constitutive expression of Cas9 is not necessary to use CRISPR in *C. albicans*, which minimizes the probability of off-target DNA modifications. The transient system also provided an opportunity to expand the experimental design by sequentially targeting multiple loci through the transformation of multiple sgRNA cassettes into one cell (Min et al., 2016).

Due to the limited number of dominant selectable markers available for use in *C. albicans*, coupling marker recycling with CRISPR-editing cassettes allow for multiple genetic editing steps to occur in the same strain. One CRISPR-based method for marker recycling is CRISPR-mediated marker excision (CRIME), in which a double-stranded break occurs within the selectable marker to trigger recombination of flanking directly-repeating sequences, and subsequent excision of the selectable marker from the genome (Huang and Mitchell, 2017). Other methods have also been developed such as the *C. albicans* LEUpOUT system and the HIS-FLP system, which enable high-efficiency, markerless homozygous CRISPR editing alongside marker recycling and removal of CRISPR (Nguyen et al., 2017). Together these methods increased the capabilities of mutant generation by enabling markerless genome editing through a single transformation.

Although these CRISPR systems were shown to be effective for *C. albicans* editing, there are still opportunities to increase their efficiency, such as through optimizing sgRNA expression and processing in the cell. For instance, testing various sgRNA promoters and post-transcriptional RNA processing schemes found that RNA transcribed under the control of the *ADH1* promoter increased CRISPR-based mutation rates 10-fold (Ng and Dean, 2017). Additionally, sgRNA flanked by a 5′ tRNA rendered a more stable mature sgRNA, lending two important improvements to CRISPR editing efficiency in *C. albicans* (Ng and Dean, 2017).

### CRISPR in Non-*albicans Candida*

After piloting the CRISPR-Cas9 gene editing system in *C. albicans*, implementation in other *Candida* species was quick to follow. While in some cases adapting previously established yeast CRISPR-Cas9 systems for non-*albicans Candida* species did not require further modifications, in most cases replacing elements of the CRISPR construct with species-specific regulators were necessary to achieve efficient genetic modifications. To avoid the additional adjustments needed to express plasmid-encoded sgRNAs and Cas9 in these species, an expression-free CRISPR-Cas9 was designed, in which purified Cas9 and CRISPR RNAs (crRNA and tracrRNA), were complexed to form ribonucleoproteins (RNPs) exogenously (Grahl et al., 2017; Morio et al., 2020). These RNPs can be transformed into diverse *Candida* species, along with a repair template for DSB repair. Compared to earlier methods that only required the use of a gene disruption construct, the addition of RNPs led to an increase in transformation efficiency in all of the clinical isolates tested, from 10 to 70% in haploid *C. lusitaniae*, 20 to 60% in *C. glabrata*, and 50 to 70% in *C. auris* (Grahl et al., 2017). The use of commercially available Cas9 and custom-designed RNA can be exploited as an expression-free system, and is beneficial to study *Candida* species where limited molecular genetic tools (plasmids, selectable markers, species-specific promoters, etc.) are available. Despite its convenience, this method is not cost-effective compared with plasmid-based expression of CRISPR-Cas9 machinery (Grahl et al., 2017; Morio et al., 2020). Therefore, in parallel to these expression-free systems, species-specific CRISPR-Cas9 systems have also been developed.

#### CRISPR in *C. glabrata*

Due to its closer evolutionary relationship to *S. cerevisiae* compared with members of the *Candida* CTG clade, CRISPR-Cas9 technology was quick to be established in the fungal pathogen *C. glabrata*, using a CRISPR-Cas9 system from *S. cerevisiae* as a foundation (DiCarlo et al., 2013; Enkler et al., 2016). Since episomal DNA is stable in *C. glabrata*, both Cas9 and sgRNA can be expressed from autonomously replicating plasmids. Two vectors expressing sgRNAs were designed to account for any species-specific regulatory requirements, one under the control of *S. cerevisiae* RNA polymerase III promoter *SNR52* and the other using *C. glabrata RNAH1* in conjunction with a Tyr 2 tRNA terminator sequence, *tTy2*. Cas9 was placed under the *C. glabrata pCYC1* promoter as a replacement for the *S. cerevisiae pTEF1* promoter, which was found to reduce fitness of its host *C. glabrata* strain (Enkler et al., 2016).

As NHEJ is the most common mechanism to repair DSBs in *C. glabrata*, these CRISPR systems used plasmids expressing Cas9 and sgRNA, but did not include a DNA repair template (Vyas et al., 2018). Using this system, with *C. glabrata*-specific promoters, efficient levels of indel mutations were observed in *C. glabrata*. To determine whether a CRISPR-Cas9 system could achieve targeted deletions via HDR in *C. glabrata*, two different gene disruption constructs, serving as DNA repair templates for HDR were transformed alongside the Cas9 and sgRNA plasmids to target the *ADE2* locus: a short repair template was used to introduce a stop codon, and a long repair template was assembled to disrupt the *C. glabrata HIS3* gene. This CRISPR-Cas9 system was able to reduce the previously recommended length of homology regions from 500 base pairs to as low as 20–200 base pairs to achieve efficient HDR and gene disruption (Schwarzmüller et al., 2014; Enkler et al., 2016).

While initial applications of the CRISPR-Cas9 technology in *C. glabrata* required the use of two expression plasmids that expressed sgRNA and Cas9 separately, the system was eventually streamlined into a single construct (Vyas et al., 2018). This Unified Solo CRISPR system contains the sgRNA, *CAS9*, and a repair template all in a single plasmid, and allows for pooled mutagenesis screens and easy recycling of the system for subsequent gene editing. Targeting *ADE2* as a proof-of-concept, this CRISPR system produced 62–71% successful *ade2* mutants that possessed the HDR repair template sequence, while the rest possessed indel mutations as a result of NHEJ (Vyas et al., 2018). Efficiency varied based on the promoter driving *CAS9* expression; using the *C. albicans pENO1* resulted in higher frequencies of NHEJ and fewer HDR events, compared to using the *S. cerevisiae pTEF1*. Recently, a modified version of the Unified Solo CRISPR-Cas9 system in *C. glabrata* was developed, to place *CAS9* under the *C. glabrata MET3* inducible promoter; this construct decoupled the transformation of the plasmid into cells from the induction of DSBs and repair mechanisms, allowing for a closer analysis of each of these steps (Zordan et al., 2013; Maroc and Fairhead, 2019).

#### CRISPR in *C. lusitaniae*

A transient CRISPR-Cas9 system has also been implemented for gene disruption in another opportunistic pathogen, *C. lusitaniae*, a haploid member of the *Candida* CTG clade (Norton et al., 2017). Here, the *C. lusitaniae* constitutive *TDH3* promoter drives expression of *CAS9* on one plasmid, while the sgRNA is expressed by another plasmid under the RNA polymerase III promoter, *pSNR52*. The repair template for this system consists of the codon-optimized drug selection marker *SAT1* (Reuss et al., 2004), flanked by either short 80-base pair or long 1,000-base pair homology regions to the target gene locus. As a proof of concept, this gene deletion method was tested on the *ADE2* gene, where short homology regions proved insufficient to delete the gene of interest. In the absence of CRISPR-Cas9, the repair template with long homology arms produced some *ade2* mutants and fairly low targeting efficiency (Norton et al., 2017). While the use of the *C. albicans*-optimized CRISPR-Cas9 system did not improve the deletion rate in diploid cells, the *C. lusitaniae*-optimized CRISPR-Cas9 system boosted the transformation and targeting efficiency by 2-fold and 20-fold, respectively, compared to the repair template used alone. The deletion percentage in the haploid strain reached 36% when edited with CRISPR-Cas9, ~4-fold higher than transformation with only the repair template and not CRISPR-Cas9 (Norton et al., 2017).

The relatively low efficiency of the CRISPR-Cas9 system in *C. lusitaniae* could partially be attributed to NHEJ events competing with HDR as a mechanism of CRISPR-based DSB repair. It was observed that haploid strains of *C. lusitaniae* lacking genes involved in NHEJ (*KU70* and *LIG4*) increased *ADE2* gene deletion frequency from 25 to 49% in the *ku70*^−/−^ mutant, and up to 81% in the *ku70*^−/−^*lig4*^−/−^ double mutant (Norton et al., 2017). Using these NHEJ mutants, target deletions were obtained at other loci, such as *UME6*, at efficiencies of up to 81%. While the *C. lusitaniae* NHEJ mutants combined with CRISPR-Cas9 machinery show promise, this model may not always be an applicable model, as impairing the NHEJ pathway is thought to reduce fungal virulence (Goins et al., 2006; Morio et al., 2020).

#### CRISPR in *C. parapsilosis, C. orthopsilosis*, and *C. metapsilosis*

Similar to the *C. glabrata* “Unified Solo” vector design, a transient plasmid-based CRISPR-Cas9 system has been developed for use in *C. parapsilosis*, another etiological agent of candidiasis (Lombardi et al., 2017). Since *C. parapsilosis* is another member of the *Candida* CTG clade, a codon-optimized CAS9 was used under the control of the *C. parapsilosis* pTEF1, along with the sgRNA, which was placed under the control of a *C. parapsilosis* RNA polymerase II promoter, *pGAPDH*, flanked by two ribozyme sequences, the hammerhead (HH), and hepatitis delta virus (HDV) ribozymes (Ng and Dean, 2017). This construct was initially piloted to insert stop codons into the *ADE2* reporter gene, with or without a repair template. In the presence of the repair template, 80–100% of resulting transformants were pink, a sample of which were confirmed to all possess the correct *ADE2* mutation, while, in the absence of the repair template, no pink colonies were obtained (Lombardi et al., 2017). The *C. parapsilosis* CRISPR-Cas9 system has proven effective in many genetic backgrounds, including clinical isolates. However, depending on the strain, the number of transformants varied greatly from 10 to 1,000, and the gene editing efficiency also varied from unsuccessful to 100%. When the repair template containing 40-base pair homology regions was present, DSB repair favored HDR, while in its absence, NHEJ was more frequent (Lombardi et al., 2017).

Overall, the CRISPR-Cas9 system in *C. parapsilosis* is very promising. *CAS9* and the selection marker are both expressed on a plasmid that can be recycled to allow for sequential gene alterations, with double mutants being achieved at an efficiency of up to 100% (Lombardi et al., 2017). Using a transient system with a drug selection marker eliminates the need to use auxotrophic strains, allowing for the manipulation of clinical isolates. The use of the RNA polymerase II promoter greatly increases the efficiency of the system, potentially by promoting higher expression levels of the sgRNA compared to the RNA polymerase III promoter. In addition to introducing genetic mutations, the system was also shown to accurately induce gene deletions and can be used for tagging genes (Lombardi et al., 2017).

Recently, an adaptation of this system was developed that simplifies the cloning protocol to insert the sgRNA sequence in between two ribozyme sequences into a single step, making it more practical for efficient cloning on numerous constructs (Lombardi et al., 2019a). In the original system, changing the guide sequence also meant changing the HH ribozyme sequence, making the process onerous if dealing with many gene targets. To circumvent this issue, HH was replaced by a *C. parapsilosis* tRNA sequence that did not need to be altered with each new guide insertion; sgRNAs were also designed with overhangs that possess SapI restriction enzyme cut sites that are compatible with its insertion site in the construct. This new design demonstrated a similar gene targeting efficiency to the original system, and also showed that short homology regions in the repair template could be used to achieve HDR (Lombardi et al., 2019a). The original CRISPR-Cas9 plasmid designed for *C. parapsilosis* was also validated as a tool to enable genome editing in two closely related species: *Candida orthopsilosis* (Zoppo et al., 2018) and *Candida metapsilosis* (Lombardi et al., 2019a).

#### CRISPR in *C. tropicalis*

Applying the same transient CRISPR-Cas9 system used in *C. parapsilosis* to *C. tropicalis*, required adjustments to the plasmid to include components that were previously confirmed to function in this clinically and industrially relevant species (Defosse et al., 2018). A new plasmid was designed that placed *CAS9* under the *Meyerozyma guilliermondii pTEF1, SAT1* under the *C. dubliniensis pTEF1*, and used a tRNA-sgRNA-ribozyme sequence, which was placed in between the *Ashbya gossypii pTEF1* and *S. cerevisiae CYC1* terminator. Restriction sites surround each of these components, allowing for alterations of almost every aspect of this construct (Lombardi et al., 2019a). Efficiency ranged from 88–100% when trying to introduce stop codons in the *ADE2* gene using an HDR repair template with 60-base pair homology arms. Similar to CRISPR editing in *C. parapsilosis*, in the absence of the repair template, NHEJ was highly efficient in *C. tropicalis* (Lombardi et al., 2019a).

Both an integrative and alternative transient CRISPR-Cas9 system were developed concurrently for use in *C. tropicalis* (Zhang et al., 2020). A number of candidate promoters were screened for efficient activity from the *C. tropicalis* genome, of which the *GAP1* and/or *FBA1* promoters were selected to express *CAS9* and the sgRNA. Since *pGAP1* and *pFBA1* are RNA polymerase II promoters, the HH and HDV ribozyme sequences flanked the sgRNA (Zhang et al., 2020). Fusion of the DNA repair templates to the CRISPR-Cas9 construct resulted in homozygous single gene mutants at an efficiency of 83–100% when targeting the reporter genes *ADE2* and *URA3*, and up to 32% for the respective double mutant. Like the first plasmid-based CRISPR-Cas9 system for *C. tropicalis*, this transient system rarely induces NHEJ events in the presence of a repair template and does not require integration of a selection marker into the genome (Zhang et al., 2020). Therefore, both CRISPR-Cas9 systems developed for *C. tropicalis* can successfully edit the genome at high efficiencies.

#### CRISPR in *C. auris*

As emergence of the pathogen *C. auris* continues to be a global concern, CRISPR-Cas9 tools have been employed to better understand the unique biology of this *Candida* species, and to identify factors that contribute to the high frequency of multidrug resistance (Jackson et al., 2019). Soon after the development of CRISPR-Cas9 in *C. albicans*, the expression-free CRISPR-Cas9 system was used to target a putative catalase gene in *C. auris* and successfully increase the efficiency of gene deletion (Grahl et al., 2017). As another member of the CTG clade, translation of technology that was previously used on other *Candida* species did not require much additional optimization. The *C. albicans* CRISPR-Cas9 machinery (Vyas et al., 2015) was used to replace the native promoter of the *HSP90* essential gene in *C. auris* with a Tet-OFF promoter to allow regulation of its expression, without requiring the use of species-specific regulators (Kim et al., 2019). CRISPR-Cas9 tools have yet to be applied extensively in this pathogen; the use of species-specific regulators and other modifications may further improve the efficiency of CRISPR-based editing in this emerging pathogen.

#### CRISPR in Industrial *Candida* Species

While the most characterized CRISPR systems exist in many medically relevant *Candida* species, CRISPR has also been applied to other members of the genus. In the field of biotechnology, CRISPR systems similar to the ones described above have been applied to species such as *Candida aaseri* and *Candida glycerinogenes* (Zhu et al., 2019; Ibrahim et al., 2020). As CRISPR-Cas9 continues to gain prominence for its streamlined metabolic engineering potential, the number of industrially-relevant *Candida* species with well-defined CRISPR systems will likely continue to increase.

### The Expansion of CRISPR Applications in *Candida* Species

**Figure 3:**
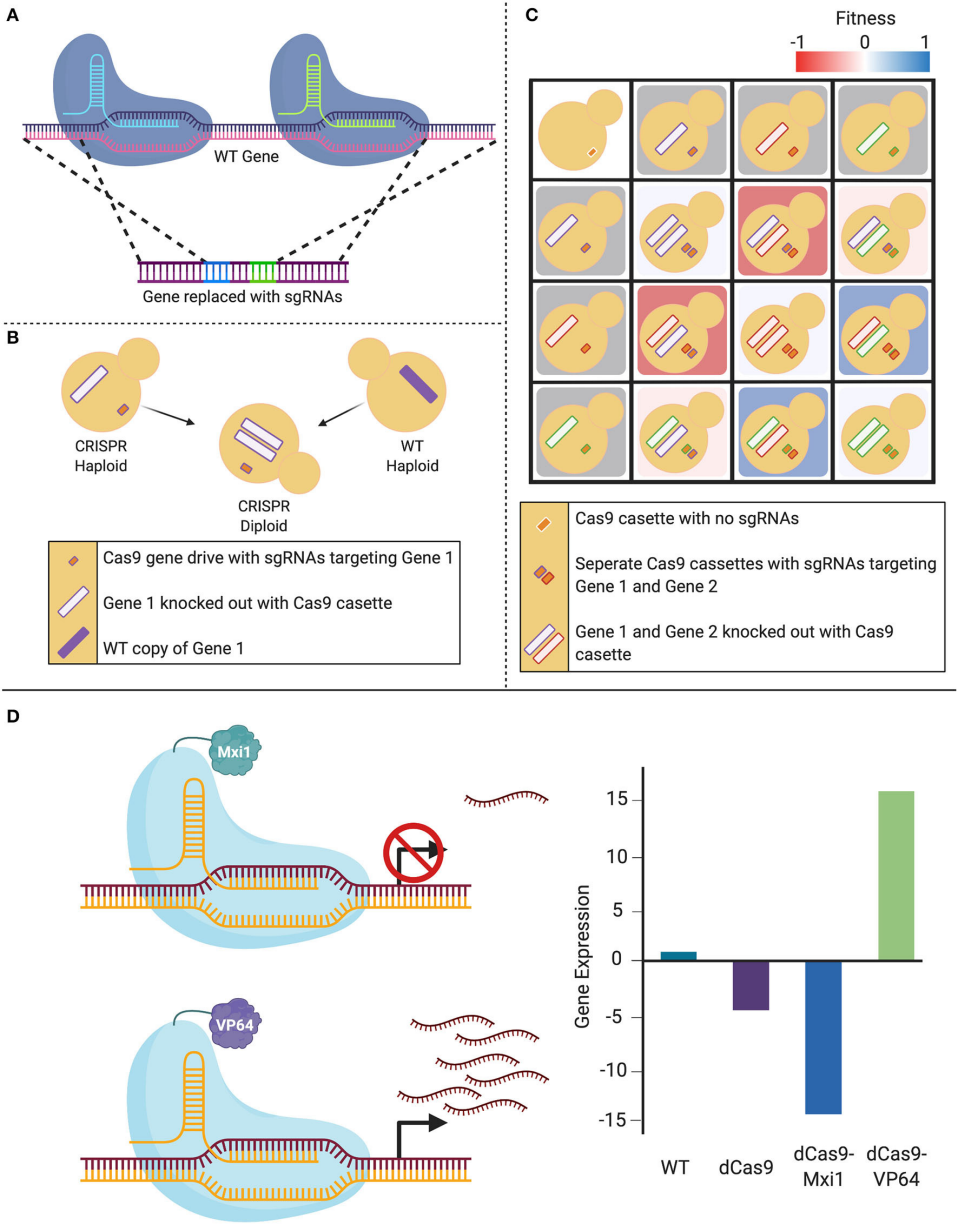
Expansion of CRISPR systems in *C. albicans*. **(A)** The CRISPR-Cas9 gene drive array leverages two sgRNAs that target upstream and downstream of the gene of interest, mediating a double-stranded break. This system then uses homology-directed repair to replace the targeted wild-type (WT) gene with a repair template that contains the sgRNAs. **(B)** When a haploid containing this CRISPR-Cas9 gene drive array is mated to a WT haploid, the WT gene is also deleted in the resultant diploids as the gene drive continues to propagate. **(C)** This system can be employed to create haploids with gene drives targeting distinct loci that can be mated to form combinatorial double-gene deletions. These double mutants can be used to assess genetic interactions when compared to either their single-mutant counterparts or the non-targeted control. **(D)** The nuclease-dead Cas9 (dCas9) can be fused to transcriptional modifiers in order to manipulate gene expression. When targeted to a gene promoter, the CRISPR-dCas9 system can reduce gene expression via steric hindrance of the DNA-dCas9 complex preventing RNA polymerase from efficiently transcribing the gene. This repression can be increased with the addition of repressors such as Mxi1 or Nrg1. Alternatively, gene expression can be increased by transcriptional activators such as VP64 that recruit RNA polymerases to the targeted gene.

The CRISPR systems described here have provided researchers with a toolkit to robustly introduce mutations in the genome of numerous *Candida* species. Given the utility and flexibility of CRISPR-based genome manipulation systems, new variants of CRISPR techniques are currently being explored to further promote efficient genetic alterations in these species (Figure 3). The novel techniques described in this section have thus far only been applied in *C. albicans*, but undoubtedly applications to other important *Candida* species will follow.

In 2018, Shapiro et al. developed a CRISPR-Cas9-based gene drive platform to rapidly generate a *C. albicans* double-gene deletion library, with applications for genetic interaction analysis (Shapiro et al., 2018b). Taking advantage of *C. albicans*' recently characterized haploid mating capabilities (Hickman et al., 2013), this system makes use of a selfish genetic element, termed the gene drive, that deletes the target gene in a haploid cell, which when mated to a wild-type haploid cell, will propagate to the remaining wild-type loci of the target gene in the resultant diploid, leading to a homozygous deletion. The technique integrates a plasmid into the *NEUT5L* locus, which contains *CAS9*, as well as two sgRNAs that are flanked by regions of homology to the target gene. Expression of the system leads to the integration of the two sgRNAs into the target gene through HDR. When this mutant strain is mated with a wild-type strain of the opposite mating type, all of the machinery required to disrupt the second wild-type allele remains present in the cell, resulting in a homozygous gene deletion. This technique can be applied to rapidly generate homozygous double-gene deletion mutants by mating strains with different target genes (Shapiro et al., 2018b). These mutants can then be compared to the parental single-gene deletion strains to identify potential genetic interactions between target genes, and ultimately start to understand complex genetic networks in this fungal pathogen (Shapiro et al., 2018b; Halder et al., 2019, 2020).

New advancements in CRISPR technologies have also led to the development of systems that are able to manipulate gene activity without directly altering the genomic sequence. These CRISPR variants were created to either repress or activate gene expression: CRISPR interference (CRISPRi) and CRISPR activation (CRISPRa), respectively. These gene regulating systems exploit the use of a nuclease-dead Cas9 (dCas9) enzyme that is produced through site-specific mutations made in the endonuclease RuvC and HNH domains of Cas9 (Qi et al., 2013). This dCas9 retains its ability to be guided by an sgRNA, but is no longer capable of inducing a DSB. Expression of transcripts from a gene can then be controlled through designing the sgRNA to target the promoter of the gene of interest, and either induce repression or over-expression through the fusion of transcriptional repressor or activator molecules to dCas9, respectively.

The first application of CRISPRi in a *Candida* species was demonstrated using two different designs for applications in *C. albicans* (Román et al., 2019a; Wensing et al., 2019). One CRISPRi design fused dCas9 to a mammalian transcriptional repressor domain, Mxi1. The dCas9-Mxi repression system was compared to dCas9 alone using quantitative reverse transcription-PCR to measure relative expression levels of the targeted gene *ADE2*. Results showed ~20 fold repression in dCas9-Mxi1 strains as compared to the ~7-fold repression in dCas9 strains (Wensing et al., 2019). This CRISPRi approach is also useful to study essential genes and was applied to repress *HSP90*, an essential molecular chaperone in *C. albicans*, and demonstrated that CRISPRi-based *HSP90* repression renders strains sensitive to antifungal agents (Wensing et al., 2019), in accordance with previous studies (Cowen and Lindquist, 2005). The second CRISPRi design implemented a dCas9 fused to Nrg1, a cognate repressor that has been shown to repress hyphal genes (Román et al., 2019a). To validate gene repression, sgRNAs were designed to guide dCas9 and dCas9-Nrg1 complexes to the catalase gene, *CAT1*, which resulted in an increased susceptibility to oxidants compared to the wild-type strain. The overall repression achieved was between 40–60%, based on a GFP repression readout (Román et al., 2019a). CRISPRi is therefore a convenient and effective genetic depletion technique for use in *C. albicans* (Román et al., 2019a; Wensing et al., 2019).

CRISPRa uses the same dCas9 element as CRISPRi, but is instead fused to transcriptional activators. Roman et al. piloted this technique in *C. albicans* by fusing the transcriptional activation domain of Gal4 or VP64 to a *C. albicans*-optimized dCas9, which resulted a 2- to 3-fold increase in expression of the targeted gene (Román et al., 2019a). This system will enable researchers to targetedly overexpress any gene of interest in the *C. albicans* genome. Together, these techniques demonstrate CRISPR's versatility beyond gene editing, providing a scalable and relatively simple alternative to previous methods used in the field, that can potentially see widespread usage across the *Candida* genus.

## Applications of CRISPR in *Candida*

**Figure 4:**
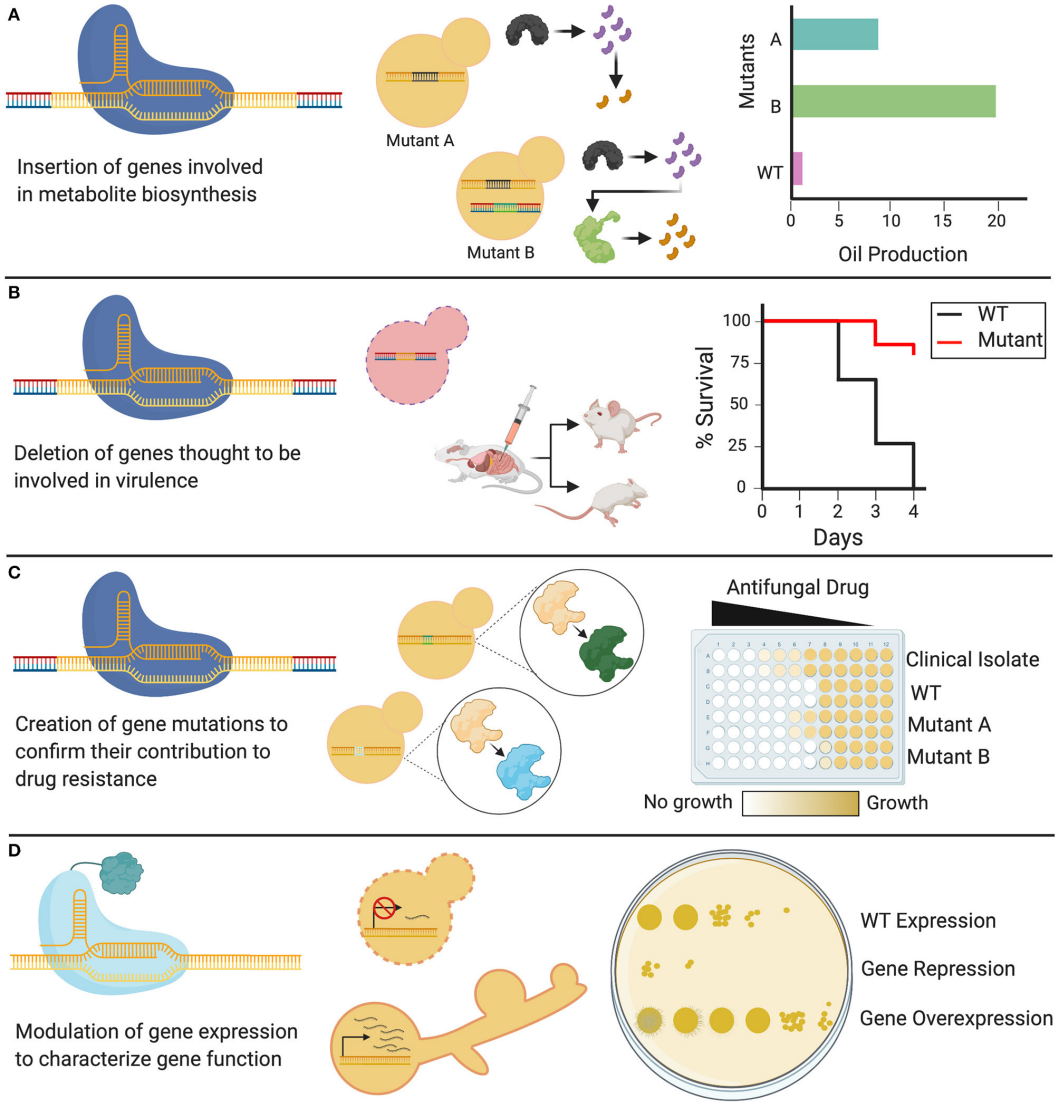
Applications of CRISPR in *Candida*. Genetic modifications induced by CRISPR systems have diverse applications in Candida species. **(A)** Cas9 can be used to knock in heterologous genes that alter WT cellular metabolism by permitting new enzymes to create new biosynthetic products. **(B)** Cas9 can be used to delete genes of interest, generating knockout mutants of non-essential genes, which can be used to identify the role of these genes in fungal virulence. **(C)** CRISPR systems can be used to introduce targeted genetic mutations through codon or base-level changes. These genetic variants can be assessed to identify the role of specific targeted mutations in resistance to antifungal drugs. **(D)** dCas9 can be used to either repress or overexpress a gene depending on the transcriptional domain fused to it, allowing for the study and phenotypic characterization of *Candida* genes, including essential genes.

As the latest addition to the gene editing toolkit, CRISPR-Cas9 technology can be used to systematically probe the biology of the *Candida* genus, from conserved traits essential in the life cycle, to species-specific characteristics. CRISPR-Cas9 can be tailored to specific applications, and can be used not only to delete single genes, but also to substitute promoters, introduce point mutations, and target gene families using a single sgRNA (Vyas et al., 2015, 2018). This versatile technology is currently being used to elucidate a diverse array of cellular processes in *Candida* species, leading to new insights on the functions of existing genes, identification of putative gene functions, and mapping epistatic interactions (Supplementary Table 1). Such biological traits can be discerned, and then implicated in applied fields of research, from the study of pathogenesis and drug resistance to the development of *Candida*-based biotechnologies (Figure 4).

### CRISPR Technologies to Study *Candida* Pathogenesis

Due to their significant global disease burden, understanding the pathogenic behavior of medically relevant *Candida* species is of the utmost importance (Lamoth et al., 2018). Since their inception, CRISPR systems have been applied to uncover the underlying mechanisms of pathogenesis in multiple *Candida* species (Vyas et al., 2015; Enkler et al., 2016; Huang and Mitchell, 2017). Mechanisms such as tissue adhesion, filamentation, hydrolytic enzyme secretion, and biofilm formation contribute to *Candida* species' ability to cause invasive infections (Sharma et al., 2019). As the best-studied *Candida* species, a large breadth of work has been concentrated on *C. albicans*, to further elucidate general *Candida* pathology. CRISPR-based reverse genetic approaches have been instrumental in investigating *Candida* virulence, and have relied on the generation and assessment of knockout mutants to decipher genetic circuitry governing processes such as adhesion, biofilm formation, and morphogenesis.

In *C. albicans*, screening mutants has identified genes and pathways involved in filamentation (Vyas et al., 2015; Hollomon et al., 2016; Mendelsohn et al., 2017; Naseem et al., 2017; Veri et al., 2018; Lu et al., 2019; Rai et al., 2019; Silao et al., 2019; Xie et al., 2020) and biofilm formation, (Huang M. Y. et al., 2019; Feng et al., 2020; Lagree et al., 2020) conceptualizing a wider genetic network that contributes to virulence. Additional studies have used CRISPR to generate multiple deletions to also uncover genetic mechanisms that play a role in *C. albicans* virulence (Huang and Mitchell, 2017; Min et al., 2018; Feng et al., 2020; Lagree et al., 2020; Williams and Lorenz, 2020). CRISPR facilitated the generation of a triple mutant through the knockout of transcriptional activator genes involved in filamentation, *BRG1* and *UME6*, in a background strain knocked down for the cell cycle kinase gene *CAK1*. Repression of *CAK1* in this strain led to cell cycle arrest, which subsequently promoted *C. albicans* filamentation, even in the absence of the two major regulators. CRISPR was therefore applied as a useful tool to link cell cycle arrest and the filamentation regulatory network, which both contribute to enhanced biofilm formation (Woolford et al., 2016).

CRISPR systems have been used to generate adhesin knockouts in multiple species, such as *C. glabrata, C. parapsilosis, C. orthopsilosis*, and *C. auris*, in order to study the role of these adhesins in fungal virulence (Enkler et al., 2016; Zoppo et al., 2018, 2020; Singh et al., 2019). CRISPR-Cas9 technologies are also particularly powerful in their application to rapidly generate higher-order mutants, which can be used to uncover complex genetic interaction networks. As an example, Zoppo et al. used CRISPR to investigate the role of the previously uncharacterized adhesin *ALS4210*, and identified a role for this adhesin in mediating *C. orthopsilosis* adhesion to host cell tissues (Zoppo et al., 2018). To further the analysis of adhesins in *C. orthopsilosis* using CRISPR, a single sgRNA was designed to target homologous regions present within all *C. orthopsilosis ALS* family adhesin genes, resulting in simultaneous knockout of the entire family of genes, and enabling the analysis of a mutant strain lacking each of its *ALS* adhesins (Zoppo et al., 2019).

Another study by Shapiro et al. leveraged the previously discussed CRISPR gene drive array to conduct a genetic interaction analysis of *C. albicans* adhesin genes (Shapiro et al., 2018b). This study comprehensively evaluated 12 adhesin genes deleted singly or in all possible combinations of double-gene deletions, resulting in a library of 144 single- and double-knockout mutants. Existing epistatic relationships between pairwise mutations were identified, contributing to defects in the mutant's ability to form biofilms on different materials (Shapiro et al., 2018b). Results showed that certain double mutants exhibited reduced biofilm formation that varied based on the material they were grown on. This study demonstrated that no single adhesin deterministically abolishes biofilm formation, and that combinations of adhesins could be targeted to impair biofilm formation (Shapiro et al., 2018b). The ability to use a CRISPR system to rapidly generate combination adhesin mutant strains greatly contributed to the feasibility of this study.

While the gene drive platform allows for rapid, high-throughput generation of mutant libraries in *C. albicans*, CRISPR-Cas9 also enables the generation of higher-order mutants beyond pairwise gene mutations. Recent work by Wijnants et al. used CRISPR-Cas9 to characterize putative sugar phosphorylation genes in this manner, demonstrating how different combinations of genes affected cell metabolism and virulence in a murine model (Wijnants et al., 2020). Double, triple, and quadruple mutants of putative *C. albicans* sugar kinase genes (*HXK1, HXK2, GLK1*, and *GLK4*), were generated using a CRISPR marker-recycling system. These four kinases were validated to perform an essential role in glycolytic transport and growth on glucose medium. Functional redundancies were uncovered between *hxk2, glk1*, and *glk4* mutants in phosphorylating glucose, and Hxk1 was found to regulate the expression of the other three kinases, establishing a hierarchy in *C. albicans*' glycolytic metabolism pathway. Given that many *Candida* adhesins are glycoproteins, glycolytic disruption often impacts virulence processes (de Groot et al., 2013). *hxk2* mutants, which demonstrated the greatest impact on both glucose and fructose phosphorylation, also had reduced adhesive abilities and were avirulent in murine models of invasive candidiasis. Meanwhile, *hxk1* mutants formed the weakest biofilms, despite comparable adhesion levels to the wild type. The reduced virulence of all the other *hxk1* mutants, given their weak biofilm formation but hyperfilamentous phenotypes, alludes to the interplay between morphogenesis and glycolytic metabolism in orchestrating a systemic infection (Wijnants et al., 2020). This is a powerful study that used higher-order mutants to decouple adherence mechanisms, robust biofilm formation, and virulence, which provides clearer insight into multiple interconnected pathways in *C. albicans*. In this case, CRISPR technologies greatly facilitated the generation of higher-order genetic mutants in *C. albicans*, in order to unravel genetic interactions mediating pathogenicity.

### CRISPR Techniques to Decipher *Candida* Host-Pathogen Interactions

CRISPR-Cas9 technologies have been employed to study *Candida* interactions with the immune system. Initial recognition of *C. albicans* as a threat to the human host is largely mediated by pattern recognition receptors of innate immune cells binding to components of the fungal cell wall. Interactions between innate receptor Dectin-1 and *C. albicans* cell wall β-glucans are critical to mount an immune response; however, *C. albicans* has evolved strategies to mask β-glucans and avoid detection (Brown and Gordon, 2001; Wheeler and Fink, 2006; Wheeler et al., 2008; Davis et al., 2014). Mutants lacking protein kinase A (PKA), iron homeostasis regulators (Ftr1 and Sef1), and phosphatidylserine synthase (Cho1) were generated by the CRISPR-Cas9 system to elucidate the signaling pathways involved in β-glucan masking, revealing that it can be triggered by host nutritional immune responses, such as iron limitation, and downstream fungal cell wall biogenesis and remodeling (Chen et al., 2019; Pradhan et al., 2019).

In addition, the deletion of genes involved in amino acid, dicarboxylic acid, and N-acetylglucosamine metabolism suggest that *C. albicans* may be using the nutritional environment within the macrophage phagosome as a signal to promote fungal survival, reduce macrophage survival, ameliorate phagosome acidity, and increase hyphal formation. Single-, double-, triple-, and quadruple-mutant strains were generated using CRISPR and used to evaluate the positive correlation between alternative carbon source metabolism and virulence (Williams and Lorenz, 2020). Other genes, such as the *C. albicans MNN4*-like gene family, were found to benefit the immune response and promote phagocytosis of fungal cells by positively regulating phosphomannan expression on the cell wall (González-Hernández et al., 2017). It is also important to note that, parallel to its introduction into the *Candida* gene editing toolbox, the CRISPR-Cas9 system has been used in mammalian cells and mouse models to elucidate host-fungal interactions; these studies have highlighted the importance of host sphingolipid biosynthesis, C-type lectin receptor crosstalk, and activation of the neutrophil/IL-17F axis to mount an immune response against *C. albicans* (Tafesse et al., 2015; Thompson et al., 2019; Bai et al., 2020; Zhou et al., 2020). These examples highlight the important role that CRISPR techniques have played in understanding host-pathogen dynamics from both the fungal and host perspectives.

### CRISPR as a Means to Probe Antifungal Drug Susceptibility and Resistance

Treatment of *Candida* infections relies on the use of antifungal drugs that target the fungal cell to either arrest growth or induce cell death (Perfect, 2017). There are three main classes of antifungals that are used for monotherapy against *Candida* infections: the fungistatic azoles and fungicidal polyenes that target the production and structure of ergosterol, respectively (Ellis, 2002; Whaley et al., 2017), and the fungicidal echinocandins that target the enzyme (1,3)-β-D-glucan synthase, needed for synthesis of the fungal cell wall (Perlin, 2015). Pyrimidines are yet another class of antifungal drugs, which target DNA synthesis, but are less favored as a monotherapy for treatment of *Candida* infections (Francois et al., 2005; Patil et al., 2015). These antifungals are effective and are broadly used to treat a variety of *Candida* infections. However, a predictable consequence of their extensive use is the subsequent rise in antifungal drug resistance across multiple *Candida* species (Cowen et al., 2014; Sharma et al., 2019). While *Candida* species have shared homology between drug resistance genes, resistance mechanisms are not always conserved between species or even within different isolates (Chen et al., 2018; Ksiezopolska and Gabaldón, 2018). Therefore, robust functional genomic and chemo-genomic profiling assays are needed to understand the molecular mechanisms mediating the resistance to antifungal drugs (Lee et al., 2020), and CRISPR can be a powerful tool to enable these studies.

CRISPR-Cas9-based platforms have helped to uncover the genetic mechanisms underlying susceptibility to all major classes of antifungals. Studies have investigated polyene susceptibility using CRISPR in *C. albicans* (Min et al., 2018; Huang C.-Y. et al., 2019) as well as probed the underlying mechanisms of azole susceptibility in *C. albicans* (Vyas et al., 2015; Liu and Myers, 2017a,b; Chen et al., 2018; Shapiro et al., 2018b; Nishimoto et al., 2019), and other *Candida* species such as *C. orthopsilosis* (de San Vicente et al., 2019; Morio et al., 2019), and the highly azole-resistant *C. auris* (Kim et al., 2019; Rybak et al., 2019, 2020). Similar studies, using mutants created with CRISPR-Cas9, have screened for genes involved in echinocandin susceptibility in *C. albicans* (Lee et al., 2018; Lagree et al., 2020) and *C. glabrata* (Hou et al., 2019). CRISPR-based studies have also been used to characterize the role of *CDC43*, the β subunit of geranylgeranyltransferase type I (GGTase I), in resistance to caspofungin in *C. albicans, C. parapsilosis*, and *C. tropicalis* (Sun et al., 2020).

Despite the complex and diverse nature of mutations involved in antifungal drug resistance, which vary greatly across clinical isolates, CRISPR-Cas9 technology has proven to be effective in elucidating complex mechanisms of resistance. In *C. lusitaniae*, a CRISPR-Cas9 gene-targeting cassette was used to introduce single base-pair modifications via HDR, to validate that the V668G substitution in the putative transcription factor *MRR1* confers resistance to both the azole fluconazole, and the pyrimidine 5-fluorocytosine (5-FC) via upregulation of the multidrug transporter *MFS7* (Kannan et al., 2019). CRISPR-Cas9 was further used to validate these results by generating an *mrr1*Δ/*mfs7*Δ double-mutant strain, which was susceptible to fluconazole and 5-FC. CRISPR-Cas9 was further employed to perform gene reversions using HDR to restore wild-type copies of the *ERG3* and *ERG4* enzymes involved in sterol biosynthesis, confirming that their deletions lead to polyene resistance (Kannan et al., 2019). Such studies aid in the discovery and confirmation of genomic alterations, ranging from single base edits to large deletions, that ultimately lead to the development of drug-resistant phenotypes.

CRISPR has also been used to validate the mechanisms of resistance to new putative antifungal agents (Kapoor et al., 2019). For example, Manogepix (MGX), an antifungal in the early stages of development, targets the glycosylphosphatidylinositol (GPI) biosynthesis enzyme Gwt1 and prevents proper cell wall synthesis. Multiple *Candida* species (*C. albicans, C. glabrata, C. parapsilosis, C. tropicalis*, and *C*. *auris*) were serially passaged in the presence of MGX to promote development of resistance, and a decrease in susceptibility to MGX was seen in all species, caused by a valine to alanine substitutions in the Gwt1 protein. CRISPR editing was used to recreate the V163A substitution in a wild-type *C. glabrata*, which resulted in reduced susceptibility to MGX, thus confirming that the valine substitution is sufficient to impart resistance (Kapoor et al., 2019). The efficiency with which CRISPR can be used to introduce mutations into diverse *Candida* species will be critical to similarly validate novel mechanisms of drug resistance to new antifungal drugs.

### Identifying Novel Antifungal Drug Targets and Vaccines Using CRISPR Technology

The CRISPR-Cas9 gene editing system can be applied to the identification of novel drug targets, whether it be to help boost the host immune response against the pathogen, or to directly inhibit the growth and survival or virulence of the pathogen itself. In addition to the signaling pathways involved in host-pathogen immune interactions, other putative drug targets have been identified using CRISPR-Cas9. By using CRISPR tools to genetically engineer *Candida* species, pathogen-specific and essential genes involved in fitness and virulence can be identified as unique drug targets, or as adjuvants for existing antifungal therapies.

Previously annotated essential genes in the model yeast, *S. cerevisiae* have homologous counterparts in *Candida* species, which can serve as a starting point in the search for novel antifungal drug targets. CRISPR-mediated deletion of *S. cerevisiae CDC8* and *CDC43* homologs in *C. albicans* and *C. glabrata*, respectively, led to severe fitness defects; in *C. albicans*, the absence of Cdc8 kinase also sensitized the strain to the polyene amphotericin B and the pyrimidine 5-FUrd, while the lack of GGTase I *CDC43* sensitized multiple *Candida* species to echinocandins (Huang C.-Y. et al., 2019; Sun et al., 2020). A novel GGTase I inhibitor, L-269289, also demonstrated fungicidal activity against *C. glabrata*, suggesting that CRISPR-based investigation of essential genes can lead to potential antifungal candidates (Huang C.-Y. et al., 2019).

In *C. albicans*, implementation of CRISPR systems have enabled the study of essential gene function through construction of conditional alleles, the introduction of regulatable promoters, or the use of CRISPRi-based repression (Vyas et al., 2015; Kim et al., 2019; Román et al., 2019a; Wensing et al., 2019). While essential genes identified in yeast are often conserved in mammalian cells, targeting pathogen-specific motifs on their respective proteins reduces opportunity for off-target effects. For example, a unique nucleotide-binding domain in *C. albicans* Hsp90 is currently being exploited for drug development (Cowen, 2013; Whitesell et al., 2019; Huang et al., 2020). CRISPRi has been used as an efficient technique to target essential genes such as *HSP90* for genetic repression, and can recapitulate phenotypes associated with loss-of-function of this essential gene (Wensing et al., 2019). This suggests that CRISPRi and other CRISPR-based repression systems will be useful tools for the study of essential genes that may serve as novel antifungal drug targets.

While there are a limited number of single gene targets available as antifungal targets, combinations of genes can be targeted in an exponentially greater number of possibilities. The creation of homozygous double-deletion mutants using the CRISPR-Cas9 gene drive platform and other systems can be a useful technique to uncover combination targets that influence fungal survival. As an example, two putative efflux pump genes, *TPO3* and *YOR1*, were both viable as single-deletion mutants, but when deleted in combination rendered the resulting double-deletion mutant non-viable (Shapiro et al., 2018b). Similarly, the *ALS3* adhesin gene was found to be involved in multiple negative genetic interactions with other adhesin genes, such as *HWP1*, under multiple biofilm growth conditions (Shapiro et al., 2018b). Simultaneously targeting the products of genes exhibiting these negative genetic interactions (or synthetic lethal interactions, in the case of *TPO3* and *YOR1*) are ideal candidates for combination therapies.

Recombinant *C. albicans* Als3p is being used as the antigen in vaccine development against various species of *Candida* as well as *Staphylococcus aureus*, with the recent phase II clinical study demonstrating the NDV-3A candidate as a promising therapeutic vaccine against vulvovaginal candidiasis (Spellberg et al., 2006; Schmidt et al., 2012; Edwards et al., 2018; Shapiro et al., 2018b). Using CRISPR-Cas9 gene-edited strains of *C. auris*, anti-sera from NDV-3A-vaccinated mice were found to be cross-reactive to the Als3p homolog in *C. auris*, inducing a potent adaptive immune response, preventing biofilm formation and enhancing killing by macrophages. Thus, CRISPR-Cas9 gene editing can elucidate antibody-epitope interactions by modifying epitopes of the target protein at the genomic level (Singh et al., 2019).

### Industrial and Environmental Applications of CRISPR in *Candida*

Aside from its clinically relevant pathogens, the *Candida* genus includes species that possess an incredible propensity to metabolize and synthesize biological compounds (Papon et al., 2012). *C. tropicalis*, for instance, can metabolize petroleum by-products and biomass feedstocks, and synthesize valuable chemicals such as ω-hydroxy fatty acids and long-chain dicarboxylic acids (Picataggio et al., 1992; Lu et al., 2010; Cao et al., 2017; Zhang et al., 2020). The CRISPR-Cas9 gene editing tool can greatly advance the application of such *Candida* species as a platform for bioproduction in industry.

A transient CRISPR-Cas9 system was used to introduce genes encoding a heterologous pathway for β-carotene synthesis from *Mucor circinelloides* into *C. tropicalis*, allowing *C. tropicalis* to serve as a molecular factory for this commercially relevant pigment (Ahmad et al., 2013; Gao et al., 2017; Zhang et al., 2020). In this manner, CRISPR-Cas9 technology can allow *Candida* to be genetically manipulated at a more rapid and efficient pace compared to non-conventional fungal species or other eukaryotes for which this technology is not yet available, and thus serve as the preferred platform to synthesize useful metabolites. The use of *C. tropicalis* and other *Candida* species to produce heterologous compounds at a productivity comparable to other industrial organisms, such as *S. cerevisiae*, still requires much optimization; foreign gene expression must be compatible with the native co-factors and regulators available in the cell. In addition, other genes in the cell must be mobilized to produce the required precursors for the system to produce biomaterials at a larger scale (Ahmad et al., 2013; Gao et al., 2017; Zhang et al., 2020).

The innate potential of several environmental species of *Candida* could also be exploited using CRISPR technology, in addition to using them as vessels for heterologous synthetic pathways. *Candida aaseri* SH14 was first isolated from the compost of oil palm; its lipolytic nature and ability to survive solely off of atypical carbon sources, such as fatty acids and alkanes, could make *C. asseri* SH14 a platform for chemical synthesis from plant oils (Picataggio et al., 1992; Ibrahim et al., 2020). CRISPR-Cas9 was employed to impair the ß-oxidation pathway in *C. asseri* SH14, to divert from fatty acid catabolism into acetyl-CoA to long-chain dicarboxylic acid production instead, as was done in the past with *C. tropicalis* using older genetic tools. The CRISPR system promoted HDR in this species and enabled multiple related genes to be disrupted with just one sgRNA that targeted conserved sequences at an efficiency of 70% (Picataggio et al., 1992; Ibrahim et al., 2020). While conversion of fatty acids to dicarboxylic acids was still slow, yield could be improved by the introduction of overexpression cassettes for rate-limiting enzymes in the process, a strategy that is currently underway using the efficient CRISPR-Cas9 tool (Picataggio et al., 1992; Ibrahim et al., 2020).

## Barriers to The Widespread Use of CRISPR in *Candida*

The development of novel CRISPR-Cas9 technologies has fueled an unprecedented efficiency and accuracy in the genome editing of *Candida* species. Despite these advantages, large-scale adoption of CRISPR use in *Candida* species may, in some cases, have been slowed by the existing wealth of other gene-editing systems for these species (Holland et al., 2014; Schwarzmüller et al., 2014; O'Meara et al., 2015; Motaung et al., 2017). For instance, several *Candida* mutant libraries are already constructed and are publicly available, and thus, many research groups continue to use these useful, pre-existing libraries and mutants for functional genomic studies (Schwarzmüller et al., 2014; Brunke et al., 2015; Ho and Haynes, 2015). While CRISPR-Cas9-mediated gene targeting is user-friendly and efficient, the creation of large-scale mutant libraries is an arduous task and still requires the transformation and selection of each mutant individually (Segal et al., 2018). Recent ventures to create mutant libraries using CRISPR-Cas9 technology may still gain momentum in the research field, as they can be used to study open reading frames that have not yet been mutated in current strain collections, especially in non-*albicans* species (Sadhu et al., 2018; Shapiro et al., 2018b; Adames et al., 2019; Lombardi et al., 2019b). The CRISPR-Cas9 system would also allow for the future creation of mutant libraries for emerging pathogens such as *C. auris*.

As a comprehensive and reliable gene editing tool, CRISPR would evidently prove useful in future clinical and commercial applications involving *Candida*, but legal battles over intellectual property ownership have led to hesitation and uncertainty amongst companies wishing to benefit from CRISPR-Cas9 technology (Sherkow, 2015; Raschmanová et al., 2018; Tripathy, 2019). In the United States, a fierce patent dispute for inventorship of CRISPR-Cas9 for eukaryotic cells recently came to a close with the University of California holding a broad patent for using CRISPR-Cas9 as a general gene editing technology, and the Broad Institute of MIT possessing a more specific patent for its use in eukaryotes. For companies seeking to use the technology in eukaryotes, it becomes unclear whether they need to obtain licenses from both parties to avoid future liability suits, in which case costs of commercialization would increase (Sherkow, 2015; Tripathy, 2019). Such broad patents may potentially lead to an increase in licensing restrictions within the market, which could even extend to academia (Sherkow, 2015; Tripathy, 2019). The legal landscape surrounding the commercialization of CRISPR-Cas9 technology in *Candida* will remain unclear until patent holders can reach a settlement and ensure technology transfer will follow progressive precedents of past innovations, to grant non-exclusive, application-specific licensing that will not unfairly impact smaller businesses (Tripathy, 2019). A solution may be on the horizon, as research groups continue to employ and modify CRISPR-Cas9 gene editing with innovative designs and new applications that exceed the scope of what patents currently cover (Ledford, 2017).

While historical and legal forces have a profound influence on the applications of CRISPR technology in *Candida* species, the technology itself also harbors some noteworthy limitations. The system relies on the induction of DSBs and the host's subsequent repair mechanisms. While the presence of a repair template promotes DSB repair via precise HDR, the level of imprecise NHEJ that occurs varies depending on the species, or even between strains of the same species. Notable examples include the low gene editing efficiency of CRISPR-Cas9 in *C. lusitaniae*, an organism that experiences a relatively high frequency of NHEJ, as well as the large range of efficiencies in *C. parapsilosis*, depending on the target gene as well as the genetic background (Lombardi et al., 2017; Norton et al., 2017). Further modifications thus had to be made depending on the species in question, which included species-specific promoters to regulate Cas9 and sgRNA expression. Ideal regulators to include in CRISPR-Cas9 constructs have yet to be identified for all *Candida* species, signaling a gap in the current *Candida* genetic toolbox (Ng and Dean, 2017; Morio et al., 2020).

The reliability of the CRISPR system also varies depending on the target locus; the exact determinants of efficiency are still to be determined for each species and could be impacted by factors such as the position and length of the template relative to the cut site, as well as sgRNA design (Román et al., 2019b). The compatibility of sgRNA secondary structure to the target locus plays a role in determining successful CRISPR targeting. Additionally, the variable chromatin structure at the locus itself may render the region inaccessible for CRISPR editing, through steric hindrance caused by nucleosome occupancy near the gene target (Horlbeck et al., 2016; Thyme et al., 2016; Adames et al., 2019). Multiple sgRNAs might have to be tested per gene to eventually optimize the systematic design of sgRNAs. In response to this issue, a number of computational tools for sgRNA have been made available, including ones that can be specifically designed to optimize efficiency and targeting in *Candida* species, such as EuPaGDT (Naito et al., 2015; Peng and Tarleton, 2015; Labun et al., 2016; Stovicek et al., 2017). Such tools facilitate ideal sgRNA design based on factors such as off-target and on-target algorithms. Unfortunately, the most specific and sophisticated of these tools are mainly available for the most commonly studied *Candida* species, such as *C. albicans, C. glabrata*, and *C. tropicalis* (Stovicek et al., 2017; Vyas et al., 2018). These resources are also mainly used to analyze the primary structure of sgRNAs and cannot yet predict interactions with target loci as it relates to secondary structure. In addition, for the CRISPR-Cas9 system to function, the cut site of the target gene, determined by the sgRNA, must be upstream to a PAM sequence, limiting the available target sequences (Doench et al., 2016; Vyas et al., 2018).

Aside from the sgRNA design, constitutive expression of Cas9 can influence off-target effects. The DSBs caused by the CRISPR-Cas9 systems can also be problematic, as research in both human cells and *Candida* species demonstrated that DSBs result in DNA damage, which can lead to cell death (Kuscu et al., 2017; Morio et al., 2020). Stable integration of the CRISPR-Cas9 system into the genome increases the likelihood of DNA damage; this risk of DNA damage is still present in transient Cas9 expression systems, but greatly reduced (Min et al., 2016). Recent findings in *C. albicans* have shown that while CRISPR-based systems can introduce off-target genomic alterations, they occur less frequently than CRISPR-free genetic manipulation transformations (Marton et al., 2020). In many plasmid-based CRISPR-Cas9 systems, the Cas9 is easily recycled via the absence of selection. However, in *C. albicans*, most CRISPR-Cas9 constructs have to be integrated into the genome to be expressed, in which case additional steps are required to recycle the selection marker, for sequential deletion of the gene, as well as the Cas9, to avoid off-target effects (Lombardi et al., 2017; Nguyen et al., 2017). Certain transient CRISPR-Cas9 expression systems, such as the one employed in *C. lusitaniae*, were developed to ensure that Cas9 and sgRNA constructs did not integrate into the genome and were eventually lost (Doench et al., 2016; Min et al., 2016; Norton et al., 2017). However, the absence of marker recycling in these systems often involves the use of multiple selection markers and/or auxotrophic strains would be required to target multiple genes (Huang and Mitchell, 2017; Lombardi et al., 2017; Nguyen et al., 2017). Both Cas9 off-target effects and/or the absence of marker recycling in many *Candida* CRISPR-Cas9 designs thus have their own limitations.

## The Future of CRISPR and *Candida*

**Figure 5:**
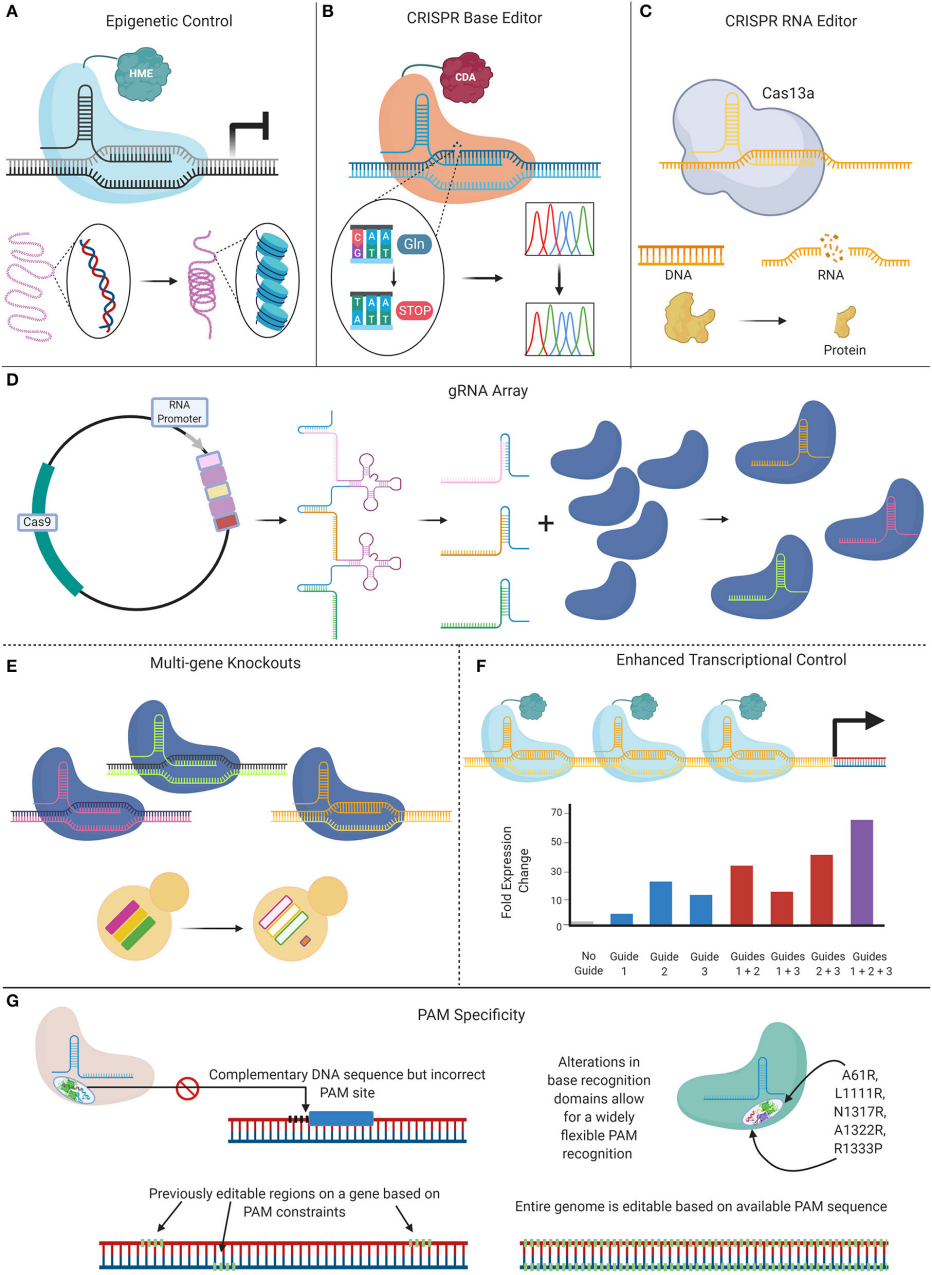
Future applications for CRISPR in *Candida*. CRISPR systems continue to evolve, and these novel technologies may be applied to *Candida* species. **(A)** CRISPR-based epigenetic modification systems can be applied to *Candida* species, whereby dCas9 is fused to histone modifying enzymes (HMEs) that can influence chromatin structure, affecting DNA expression. **(B)** Another novel CRISPR application for *Candida* species are CRISPR base editors that have a nickase variant of Cas9 fused to a cytosine deaminase that can edit DNA at a base-level resolution allowing for mutations such as premature stop codons or gene variants. **(C)** Cas variants such as Cas13a can be used to directly edit RNA molecules resulting in non-functional proteins while the genome is left intact. Improvements to existing CRISPR-Cas9 systems rely on altering either the guide RNA targeting **(D–F)** or decreasing the specificity of the PAM recognition site **(G)**. **(D)** The development of Candida-specific guide RNA arrays that have guide sequences interspaced between self-cleaving tRNA sequences, would allow for the rapid assembly of multiple guides binding to individual Cas9 proteins for further genome engineering. **(E)** These Cas9 proteins can then be uniquely targeted to multiple genes within one mutant that contains a single Cas9 cassette, efficiently creating multi-gene knockouts. **(F)** dCas9 CRISPR systems can be targeted to multiple regions in close proximity to amplify their effect on gene transcription by either repressing or overexpressing (not depicted) the targeted gene. **(G)** The PAM binding site of Cas9 can also be mutated to permit a wider range of DNA binding sites that have the potential to make any region in the genome editable, rather than being constrained by the existing genetic sequences.

Since its debut in 2015, CRISPR-Cas9 technology continues to revolutionize the potential to perform genomic interrogations in *Candida* species, from incorporating specific genetic mutations, to allowing the efficient, high-throughput generation of mutant libraries. New CRISPR-based tools are on the horizon, as the full capabilities of this technology on *Candida* research have yet to be realized. New innovations are being developed that seek to address the limitations of current CRISPR-Cas9 techniques, including genome instability caused by DSBs, PAM specificity requirements, sgRNA selection, and off-target effects (Figure 5). Future *Candida* studies can take advantage of these novel tools that currently aim to improve the efficiency of CRISPR tools by offsetting some of their limitations, while broadening their applications.

### Improvements on CRISPR-Cas Systems for Functional Genomic Analysis in *Candida* Species

Given the potential for Cas9 to induce off-target effects and DNA damage, many methods for increasing the utility of CRISPR systems rely on altering the Cas9 endonuclease (Min et al., 2016). As described, dCas9-based CRISPR systems have been used as part of CRISPRi and CRISPRa systems in *C. albicans* (Román et al., 2019a; Wensing et al., 2019), and other dCas9 fusions could be similarly used for epigenetic silencing in *Candida* species (Gjaltema and Rots, 2020). Other emerging techniques, such as CRISPR-based RNA editing (Jing et al., 2018) and base editing (Després et al., 2020) could have exciting applications in *Candida* species, to enable targeted manipulation of RNA transcripts without DNA edits, and targeted DNA nucleotide substitutions in the absence of DSBs, respectively.

The versatility of CRISPR platforms in *Candida* species allows for potential applications in multiplexed genome editing, whereby multiple genes can be targeted in a single transformation. In *S. cerevisiae*, multi-gene CRISPR perturbations are typically accomplished with the expression of multiple guides on a single RNA transcript. Once transcribed, tRNA- or ribozyme-based self cleavage allows the transcript to be processed into individual guide RNAs (Zhang Y. et al., 2019). Once each of the guides are processed into sgRNAs, they bind to Cas9 and form individual CRISPR-Cas9 complexes that can edit the genomic region complementary to the guide. This technology has been demonstrated to successfully disrupt amino acid biosynthesis genes in *C. tropicalis*, suggesting its potential to be adopted in other *Candida* species (Zhang et al., 2020). sgRNA multiplexing will expedite the rate with which higher-order *Candida* mutants can be created, and enable the delineation of complex genetic circuitry. Another benefit of sgRNA multiplexing is to bolster current CRISPRi/CRISPRa systems to perform “promoter tiling.” Here, instead of targeting different loci across the genome, multiple sgRNAs would target different regions along a single promoter. For researchers, this would allow for the identification of regions that lead to maximal gene repression or activation, enabling more precise control of relevant genes of interest (McCarty et al., 2020).

To further improve current CRISPR-Cas9 designs, modifying the flexibility of Cas9 to recognize PAM sequences beyond the canonical “NGG” sequence, can widen the genomic scope of sequences that can be modified. For CRISPRi, CRISPRa, and CRISPR base editing in particular, increasing the number of sequences available for targeting results in more precise transcriptional control or a larger number of protein variants that can be introduced. Given that the only Cas protein employed in any *Candida* strain thus far has been Cas9, the field would further benefit from exploring the implementation of other CRISPR-Cas systems, such as Cas12, already tested in other fungal organisms (Ouedraogo and Tsang, 2020). Furthermore, much research has investigated alternative Cas proteins and their PAM requirements, which could also be optimized for *Candida* species (Gleditzsch et al., 2019). In addition to the discovery and optimization of new Cas proteins, another promising option is to create a modified Cas9 variant with a more flexible PAM recognition site. Walton et al. recently created a Cas9 mutant that has a PAM recognition sequence of “NR/YN” (where N is any nucleotide and R is A/G and Y is C/T), which can target virtually any site within a given genome. This SpRY-Cas9 was created via amino acid modifications that alter base recognition domains in the Cas9 complex, allowing the specific PAM nucleotide requirements to be relaxed (Walton et al., 2020). This tool will surely reimagine the limits of sgRNA designs for almost every organism and CRISPR system currently in use. Application of these tools would give *Candida* researchers unprecedented access to sieve through the functional genomics of this genus.

### Future Applications of CRISPR in *Candida* Species in Industry

As the CRISPR-Cas9 arsenal for *Candida* genetic engineering continues to develop, it will become an invaluable tool to unearth the hidden utility of this genus outside of the clinical setting. Members of the *Candida* genus have historically served as models for “white biotechnology,” due to their ability to convert biomass to produce antibiotics, food additives, and biodegradable alternatives to petroleum-based synthetics (Frazzetto, 2003; Papon et al., 2012). Their use in biotechnology not only stems from their ability to metabolize C_5_ sugars, but also from their applications in biological control. Some yeasts, such as *Candida guilliermondii* and *Candida oleophila*, are used to prolong plant-based food shelf-life by inhibiting growth of other fungi involved in food spoilage (Akinterinwa et al., 2008; Sundh and Melin, 2011; Papon et al., 2012). Out of 74 microorganisms isolated from oil-contaminated soils and compost, *Candida catenulata* was the easiest to culture *in vitro*, and demonstrated the highest efficiency in emulsifying and degrading petroleum hydrocarbons. Bioaugmentation of oil-contaminated soil with *C. catenulate* and food waste saw ~80% petroleum hydrocarbon degradation in 13 days, suggesting that this organism may be beneficial in future applications of compost-based bioremediation methods (Joo et al., 2008). Similarly, a strain of *C. albicans* demonstrated the rare capacity to catabolize both formaldehydes and phenolic pollutants released into soil and water from industrial waste (Tsai et al., 2005). These examples highlight how *Candida* species are suitable models to decipher the complexities behind bioconversions and metabolite synthesis, which can be translated toward environmental sustainability. The versatility of CRISPR-Cas9 technology will allow for routine genetic engineering of a wider breadth of such species, to both investigate and enhance their relevance to industrial biotechnology, and also elucidate the molecular machineries involved (Donohoue et al., 2018). CRISPR-based genetic modifications are currently being exploited in diverse microbial organisms, including cyanobacteria, actinomycetes, filamentous fungi, and numerous other microbial species, translating into a breadth of exciting industrial applications (Sun et al., 2018; Li et al., 2019; Zhang S. et al., 2019; Ouedraogo and Tsang, 2020).

## Conclusion

Given the polyphyletic nature of the *Candida* genus and the resulting roles of these species as part of contemporary human society, it is critical to have genetic tools that allow for detailed species profiling. CRISPR's widespread adoption in *Candida* species, has rapidly permitted the generation and evaluation of important mutants in the lab. These research endeavors establish foundations that translate into advances in both clinical and bio-industrial settings. Future research on the application of CRISPR technologies to other *Candida* species will continue to provide even more advantages for the global economy and human health, ultimately leading to a more comprehensive understanding of this multifaceted genus.

## Author Contributions

DU, JS, LW, and RS each contributed to conceptualization and editing of the manuscript. DU, JS, and LW wrote the manuscript and generated the figures and tables. All authors contributed to the article and approved the submitted version.

## Conflict of Interest

The authors declare that the research was conducted in the absence of any commercial or financial relationships that could be construed as a potential conflict of interest.
