# Supplementary material for: CRISPR-Based Genetic Manipulation of Candida Species: Historical Perspectives and Current Approaches
Source: Front Genome Ed. 2021 Jan 8;2:606281. doi: 10.3389/fgeed.2020.606281 (PMC8525362; doi:10.3389/fgeed.2020.606281)
Supplement: Supplementary file 1 [file Table_1.DOCX]

**Supplementary Table 1. A Summary of CRISPR Use In Pathogenic *Candida* spp.**

| **Organism** | **Relevance** | **Gene** | **Technique** | **Discovery** | **Reference** |
| --- | --- | --- | --- | --- | --- |
| *C. albicans* | Pathobiology | *SNF1* | HDR K81R mutation | Hyphal formation inhibited | [(Vyas et al., 2015)](https://paperpile.com/c/CEBw8c/pfPm) |
| *C. albicans* | Pathobiology | *UME6* | HDR to insert premature STOP codons | Hyphal formation inhibited  Virulence in a larvae model reduced | [(Evans et al., 2018)](https://paperpile.com/c/CEBw8c/CkGo) |
| *C. albicans* | Pathobiology | *HXL1/HXL2*  *HXK1/GLK1/GLK4* | HDR-coupled with CRISPR recycling to knockout multiple genes | Biofilm formation impaired  Virulence in a murine model reduced | [(Wijnants et al., 2020)](https://paperpile.com/c/CEBw8c/D3ij7) |
| *C. albicans* | Pathobiology | *HXK2/GLK1/GLK4* | HDR-coupled with CRISPR recycling to knockout multiple genes | Adhesion reduced  Avirulent in a murine model | [(Wijnants et al., 2020)](https://paperpile.com/c/CEBw8c/D3ij7) |
| *C. albicans* | Pathobiology | *HXK1/HXK2/GLK1/GLK4* | HDR-coupled with CRISPR recycling to knockout multiple genes | Adhesion reduced    Biofilm formation impaired    Avirulent in a murine model | [(Wijnants et al., 2020)](https://paperpile.com/c/CEBw8c/D3ij7) |
| *C. albicans* | Pathobiology | *BRG1/UME6/CAK1-*DX | HDR-guided *NAT* cassette replacement of the gene | Retained biofilm competency  Retained hyphal formation competency | [(Woolford et al., 2016)](https://paperpile.com/c/CEBw8c/Oi42K) |
| *C. albicans* | Pathobiology | 12 adhesin genes multiplexed to generate 144 mutants | HDR-guided gene-drive array replacement | Biofilm formation on a variety of medical-grade material reduced overall | [(Shapiro et al., 2018)](https://paperpile.com/c/CEBw8c/800lv) |
| *C. albicans* | Pathobiology | *RIM101* | HDR-guided *NAT* cassette replacement of the gene | Elucidated genes under the pH-dependent control of *Rim101*  Validated that the cAMP pathway can also respond to pH change despite the absence of *Rim101* | [(Hollomon et al., 2016)](https://paperpile.com/c/CEBw8c/vD3UV) |
| *C. albicans* | Pathobiology | *UME6* | HDR S436A mutation | Identified a critical phosphorylation site required for hyphal growth under hypoxia | [(Lu et al., 2019)](https://paperpile.com/c/CEBw8c/KOkyz) |
| *C. albicans* | Pathobiology | *RAD23* | HDR-guided *HIS3* cassette replacement of the gene | Biofilm formation increased  Virulence in murine model reduced  Survival in macrophages reduced  Virulence associated genes *CEF3*, *RBT4*, and *SUN41* were downregulated | [(Feng et al., 2020b)](https://paperpile.com/c/CEBw8c/cY81u) |
| *C. albicans* | Pathobiology | *RAD4* | HDR-guided *HIS3* cassette replacement of the gene | Biofilm formation increased  Virulence associated genes *CEF3* and *RBT4* were downregulated  Virulence associated gene *SUN41* was upregulated | [(Feng et al., 2020b)](https://paperpile.com/c/CEBw8c/cY81u) |
| *C. albicans* | Pathobiology | *NGS1/HXK1* | HDR-guided *NAT* cassette replacement of the gene | Hyphal formation inhibited | [(Naseem et al., 2017)](https://paperpile.com/c/CEBw8c/VUTou) |
| *C. albicans* | Pathobiology | *MIG1* | HDR-guided *NAT* cassette replacement of the gene | Hyphal formation slightly inhibited | [(Lagree et al., 2020)](https://paperpile.com/c/CEBw8c/SDkdo) |
| *C. albicans* | Pathobiology | *MIG1/MIG2* | HDR-guided *NAT* cassette replacement of the gene | Hyphal formation inhibited  Biofilm formation reduced | [(Lagree et al., 2020)](https://paperpile.com/c/CEBw8c/SDkdo) |
| *C. albicans* | Pathobiology | *NDT80* | HDR-coupled with CRISPR recycling to knockout multiple genes | Hyphal formation and cell separation when grown on serum and GlcNAc were defective | [(Min et al., 2018)](https://paperpile.com/c/CEBw8c/FSuug) |
| *C. albicans* | Pathobiology | *RON1* | HDR-coupled with CRISPR recycling to knockout multiple genes | Hyphal formation unimpacted | [(Min et al., 2018)](https://paperpile.com/c/CEBw8c/FSuug) |
| *C. albicans* | Pathobiology | *NDT80/RON1* | HDR-coupled with CRISPR recycling to knockout multiple genes | Hyphal formation and cell separation when grown on serum were defective  Hyphal formation on GlcNAc was slightly defective | [(Min et al., 2018)](https://paperpile.com/c/CEBw8c/FSuug) |
| *C. albicans* | Pathobiology | *NDT80/RON1/REP1* | HDR-coupled with CRISPR recycling to knockout multiple genes | Hyphal formation and cell separation when grown on serum and GlcNAc were defective | [(Min et al., 2018)](https://paperpile.com/c/CEBw8c/FSuug) |
| *C. albicans* | Pathobiology | *HGC1* | HDR-guided *NAT* cassette replacement of the gene | *UME6* was degraded at a slower rate  *UME6* expression was reduced | [(Mendelsohn et al., 2017)](https://paperpile.com/c/CEBw8c/uGOew) |
| *C. albicans* | Pathobiology | *PUT1*  *PUT2*  *PUT3*  *PUT1/PUT2* | HDR-guided *NAT* cassette replacement of the gene | Hyphal formation inhibited | [(Silao et al., 2019)](https://paperpile.com/c/CEBw8c/rGNi1) |
| *C. albicans* | Pathobiology | *BRG1/EFG1* | HDR with CRISPR and *NAT* marker recycling | Biofilm formation inhibited across multiple *Candida albicans* clinical isolates  Hyphal formation inhibited | [(Huang et al., 2019b)](https://paperpile.com/c/CEBw8c/Ftksw) |
| *C. albicans* | Pathobiology | *UME6/BCR1* | HDR-coupled with CRISPR and NAT marker recycling | Various degrees of biofilm and hyphal inhibition across multiple *Candida albicans* clinical isolates | [(Huang et al., 2019b)](https://paperpile.com/c/CEBw8c/Ftksw) |
| *C. albicans* | Pathobiology | *HSF1* | HDR-guided TetO cassette replacement of the promoter  This is to cause overexpression when not Tet is added or repression when Tet is added | In both cases, filamentation occurred in the absence of any inducing cues | [(Veri et al., 2018)](https://paperpile.com/c/CEBw8c/sZCqc) |
| *C. albicans* | Pathobiology | *HHT2/HHT21* | HDR-guided *NAT* cassette replacement of the gene | Viable however, doubling time significantly increased  Cell morphology elongated | [(Rai et al., 2019)](https://paperpile.com/c/CEBw8c/zzRF5) |
| *C. albicans* | Pathobiology | *Combinatorial deletion of STP2, JEN1, JEN2, HXK1, NAG1, and DAC1* | HDR-guided *SAT* cassette replacement of the gene | Cell wall morphology altered  Filamentation slightly inhibited    Survival in macrophages decreased  Virulence in murine model reduced | [(Williams and Lorenz, 2020)](https://paperpile.com/c/CEBw8c/hixrN) |
| *C. glabrata* | Pathobiology | *YPS11* | NHEJ to create premature STOP codons | Cell wall composition unaffected  Doubling time increased  Virulence in Drosophila model reduced | [(Enkler et al., 2016)](https://paperpile.com/c/CEBw8c/iZ4XV) |
| *C. glabrata* | Pathobiology | *VPK1* | NHEJ to create premature STOP codons and HDR to insert a *HIS3* cassette | Cell wall composition unaffected  Virulence in Drosophila model reduced | [(Enkler et al., 2016)](https://paperpile.com/c/CEBw8c/iZ4XV) |
| *C. parapsilosis* | Pathobiology | *ALS4770* | HDR-guided repair that introduces a premature STOP codon | Biofilm formation reduced  Adherence to human cells reduced  Colonization in vaginal murine model reduced | [(Zoppo et al., 2020)](https://paperpile.com/c/CEBw8c/rYBkW) |
| *C. parapsilosis* | Pathobiology | *ALS4780* | HDR-guided repair that introduces a premature STOP codon | Colonization in vaginal murine model reduced | [(Zoppo et al., 2020)](https://paperpile.com/c/CEBw8c/rYBkW) |
| *C. parapsilosis* | Pathobiology | *ALS4770/ALS4780* | HDR-guided repair that introduces premature STOP codons | Morphology is elongated despite the absence of inducing cues  Biofilm formation enhanced  Adherence to human cells increased  Colonization in vaginal murine model reduced | [(Zoppo et al., 2020)](https://paperpile.com/c/CEBw8c/rYBkW) |
| *C. orthopsilosis* | Pathobiology | *ALS4210* | HDR-guided NAT cassette replacement of the gene | Reduced adherence to human cells | [(Zoppo et al., 2018)](https://paperpile.com/c/CEBw8c/HKQir) |
| *C. orthopsilosis* | Pathobiology | *ALS800/ALS4210/ALS4220* | HDR-guided repair that introduces premature STOP codons | Filamentation inhibited  Reduced adherence to human cells | [(Zoppo et al., 2019)](https://paperpile.com/c/CEBw8c/R9XLS) |
| *C. auris* | Pathobiology | *ALS3* homologues*:*  *PIS50650.1*  *PIS50263.1*  *XP_018167572.2* | HDR-guided *NAT* cassette replacement of the gene | Binding to anti-Als3p IgG antibodies reduced | [(Singh et al., 2019)](https://paperpile.com/c/CEBw8c/ISUbc) |
| *C. auris* | Pathobiology | *HSP90* | HDR-guided TetO cassette replacement of the promoter  This is to cause overexpression when not Tet is added or repression when Tet is added | Filamentous growth when gene is repressed | [(Kim et al., 2019)](https://paperpile.com/c/CEBw8c/tvbMO) |

| **Organism** | **Relevance** | **Gene** | **Technique** | **Discovery** | **Reference** |
| --- | --- | --- | --- | --- | --- |
| *C. albicans* | Drug Response | *CDR1/CDR2* | HDR-guided repair that introduces a premature STOP codon | Increased fluconazole sensitivity | [(Vyas et al., 2015)](https://paperpile.com/c/CEBw8c/pfPm) |
| *C. albicans* | Drug Response | *MIG1* | HDR-guided *NAT* cassette replacement of the gene | Increased caspofungin sensitivity | [(Lagree et al., 2020)](https://paperpile.com/c/CEBw8c/SDkdo) |
| *C. albicans* | Drug Response | *MIG1/MIG2* | HDR-guided *NAT* cassette replacement of the gene | Caspofungin hypersensitivity | [(Lagree et al., 2020)](https://paperpile.com/c/CEBw8c/SDkdo) |
| *C. albicans* | Drug Response | *NDT80/RON1/REP1* | HDR with CRISPR recycling to knockout multiple genes | Increased amphotericin B sensitivity  Fungal tolerance to fluconazole abolished | [(Min et al., 2018)](https://paperpile.com/c/CEBw8c/FSuug) |
| *C. albicans* | Drug Response | 10 efflux genes multiplexed to generate 100 mutants | HDR-guided gene-drive array replacement | Overall increased susceptibility to a variety of antifungal agents | [(Shapiro et al., 2018)](https://paperpile.com/c/CEBw8c/800lv) |
| *C. albicans* | Drug Response | *PAA11* | HDR-guided repair that introduces a premature STOP codon | Increased fluconazole and posaconazole sensitivity | [(Chen et al., 2018)](https://paperpile.com/c/CEBw8c/cwxQo) |
| *C. albicans* | Drug Response | *CDR1* | HDR-guided *SAT* cassette replacement of the gene | Increased fluconazole sensitivity  *CDR2* and *RTA3* contributed to fluconazole resistance independently of the *Tac1*-Mediator Tail complex | [(Liu and Myers, 2017b)](https://paperpile.com/c/CEBw8c/kZEWx) |
| *C. albicans* | Drug Response | *MDR1* | HDR-guided *SAT* cassette replacement of the gene | Increased fluconazole sensitivity  *MRR1* increased fluconazole resistance by acting on factors other than *MDR1*  Fluconazole resistance emerged independently of the kinase, *SSN3*, and the chromatin remodeler *SNF2* | [(Liu and Myers, 2017a)](https://paperpile.com/c/CEBw8c/tBaI4) |
| *C. albicans* | Drug Response | *MRR2* | DSB at the *ADH1* locus to allow for HDR for gene insertion of *MRR2* variants | The variable contribution of different *MRR2* mutations from clinical isolates as they contribute to azole resistance | [(Nishimoto et al., 2019)](https://paperpile.com/c/CEBw8c/1FAUz) |
| *C. albicans* | Drug Response | *FKS1* | HDR guided repair to generate S645P mutations | Increased susceptibility to caspofungin  Decreased susceptibility to poacic acid | [(Lee et al., 2018)](https://paperpile.com/c/CEBw8c/Hgp4A) |
| *C. albicans* | Drug Response | *CDC8* | HDR resulting in the 36 bp deletion of the Ca-loop | Increased susceptibility to fluoropyrimidines  Increased susceptibility to amphotericin B | [(Huang et al., 2019a)](https://paperpile.com/c/CEBw8c/a46dk) |
| *C. albicans* | Drug Response | *RHO1* | HDR-guided repair to generate L198A mutation | Increased susceptibility to caspofungin | [(Sun et al., 2020)](https://paperpile.com/c/CEBw8c/69R8G) |
| *C. albicans* | Drug Response | *FKS1* | HDR-guided repair to generate F641S/S645F mutation | Decreased susceptibility to caspofungin, which is restored when treated with L-269289  Increased survival of murine models when treated with caspofungin and L-269289 | [(Sun et al., 2020)](https://paperpile.com/c/CEBw8c/69R8G) |
| *C. orthopsilosis* | Drug Response | *ZCF29* | HDR-guided repair that introduces a premature STOP codon | Increased susceptibility to caffeine  Increased susceptibility to ketoconazole | [(de San Vicente et al., 2019)](https://paperpile.com/c/CEBw8c/1plUg) |
| *C. orthopsilosis* | Drug Response | *ERG11* | HDR-guided repair to generate G458S mutation | Decreased susceptibility to fluconazole in a previously azole-sensitive isolate  Decreased susceptibility to voriconazole in a previously azole-sensitive isolate | [(Morio et al., 2019)](https://paperpile.com/c/CEBw8c/Cf31l) |
| *C. auris* | Drug Response | *CDR1* | HDR-guided *SAT* cassette replacement of the gene | Increased susceptibility to a variety of azoles | [(Rybak et al., 2019)](https://paperpile.com/c/CEBw8c/EEWfV) |
| *C. auris* | Drug Response | *MDR1* | HDR-guided *SAT* cassette replacement of the gene | Slight increased susceptibility to a variety of azoles | [(Rybak et al., 2019)](https://paperpile.com/c/CEBw8c/EEWfV) |
| *C. auris* | Drug Response | *TAC1B* | HDR-guided repair to generate A640V mutation | Increased resistance to fluconazole, mediated by an overexpression of *CDR1* | [(Rybak et al., 2020)](https://paperpile.com/c/CEBw8c/yjoBR) |
| *C. auris* | Drug Response | *HSP90* | HDR-guided TetO cassette replacement of the promoter  This is to cause overexpression when not Tet is added or repression when Tet is added | Increased susceptibility to fluconazole when *HSP90* is repressed | [(Kim et al., 2019)](https://paperpile.com/c/CEBw8c/tvbMO) |
| *C, glabrata* | Drug Response | *FKS2* | HDR-guided repair to generate E665K mutation | Reduced susceptibility to echinocandins | [(Hou et al., 2019)](https://paperpile.com/c/CEBw8c/PBeEM) |
| *C, glabrata* | Drug Response | *GWT1* | HDR-guided repair to generate V163A mutation | Reduced susceptibility to Manogepix | [(Kapoor et al., 2019)](https://paperpile.com/c/CEBw8c/DYaC0) |
| *C lusitaniae* | Drug Response | *MRR1* | HDR-guided *SAT* cassette replacement of the gene | Increased fluconazole susceptibility in drug-resistant isolate  Increased 5-fluorocytosine susceptibility in drug-resistant isolate  Decreased transcript levels of *MFS7* indicate involvement in gene regulation | [(Kannan et al., 2019)](https://paperpile.com/c/CEBw8c/6wvKE) |
| *C lusitaniae* | Drug Response | *MRR1* | HDR-guided repair to generate V668G mutation | Increased fluconazole resistance in fluconazole-susceptible strain  Increased 5-fluorocytosine resistance in 5-fluorocytosine-susceptible strain | [(Kannan et al., 2019)](https://paperpile.com/c/CEBw8c/6wvKE) |
| *C lusitaniae* | Drug Response | *MFS7* | HDR-guided *SAT* cassette replacement of the gene | Increased fluconazole susceptibility in drug-resistant isolate  Increased 5-fluorocytosine susceptibility in drug-resistant isolate | [(Kannan et al., 2019)](https://paperpile.com/c/CEBw8c/6wvKE) |
| *C lusitaniae* | Drug Response | *ERG3* | HDR-guided *ERG3* restoration | Restored amphotericin B susceptibility to wild-type levels | [(Kannan et al., 2019)](https://paperpile.com/c/CEBw8c/6wvKE) |
| *C lusitaniae* | Drug Response | *ERG4* | HDR-guided *ERG4* restoration | Restored amphotericin B susceptibility to wild-type levels | [(Kannan et al., 2019)](https://paperpile.com/c/CEBw8c/6wvKE) |
| *C. tropicalis* | Drug Response | *CDC43* | HDR-guided repair that introduces a premature STOP codon | Increased susceptibility to caspofungin | [(Sun et al., 2020)](https://paperpile.com/c/CEBw8c/69R8G) |
| *C. parapsilosis* | Drug Response | *CDC43* | HDR-guided repair that introduces a premature STOP codon | Increased susceptibility to caspofungin | [(Sun et al., 2020)](https://paperpile.com/c/CEBw8c/69R8G) |

| **Organism** | **Relevance** | **Gene** | **Technique** | **Discovery** | **Reference** |
| --- | --- | --- | --- | --- | --- |
| *C. albicans* | General Biology | *SNF1* | HDR K81R mutation | Colonies presented wrinkled morphology  Growth on maltose inhibited  Increased cold sensitivity | [(Vyas et al., 2015)](https://paperpile.com/c/CEBw8c/pfPm) |
| *C. albicans* | General Biology | *SNF1* | HDR-guided *MAL2* cassette insertion of the promoter  This causes expression of the gene only on maltose media | Verified that *SNF1* is an essential gene | [(Vyas et al., 2015)](https://paperpile.com/c/CEBw8c/pfPm) |
| *C. albicans* | General Biology | *DCR1* | HDR-guided repair that introduces a premature STOP codon | Growth at 16°C inhibited | [(Vyas et al., 2015)](https://paperpile.com/c/CEBw8c/pfPm) |
| *C. albicans* | General Biology | *HXL1/HXL2* | HDR with CRISPR recycling to knockout multiple genes | Glucose phosphorylation reduced  Growth on fructose media inhibited  Growth in glucose media slightly reduced | [(Wijnants et al., 2020)](https://paperpile.com/c/CEBw8c/D3ij7) |
| *C. albicans* | General Biology | *HXK2*  *GLK1*  *GLK4* | HDR with CRISPR recycling to knockout multiple genes | Glucose phosphorylation reduced  Growth in glucose media reduced | [(Wijnants et al., 2020)](https://paperpile.com/c/CEBw8c/D3ij7) |
| *C. albicans* | General Biology | *HXK2/GLK1/GLK4*  *HXK1/HXK2/GLK1/GLK4* | HDR with CRISPR recycling to knockout multiple genes | Glucose phosphorylation inhibited  Growth on glucose media inhibited | [(Wijnants et al., 2020)](https://paperpile.com/c/CEBw8c/D3ij7) |
| *C. albicans* | General Biology | *RAD23* | HDR-guided *HIS3* cassette replacement of the genes | Nuclear segregation decreased  Genome stability reduced  Sensitivity to UV increased | [(Feng et al., 2020b)](https://paperpile.com/c/CEBw8c/cY81u) |
| *C. albicans* | General Biology | *RAD23* | HDR-guided *MET3* cassette replacement of the promoter  This causes gene overexpression | Sensitivity to UV increased | [(Feng et al., 2020b)](https://paperpile.com/c/CEBw8c/cY81u) |
| *C. albicans* | General Biology | *NGS1/HXK1* | HDR-guided *NAT* cassette replacement of the gene | Growth on GlcNAc permitted | [(Naseem et al., 2017)](https://paperpile.com/c/CEBw8c/VUTou) |
| *C. albicans* | General Biology | *MIG1/MIG2* | HDR-guided *NAT* cassette replacement of the gene | Validated gene function downstream of the *SNF1* pathway | [(Lagree et al., 2020)](https://paperpile.com/c/CEBw8c/SDkdo) |
| *C. albicans* | General Biology | *HHT2/HHT21* | HDR-guided *NAT* cassette replacement of the gene | Cell morphology elongated  Validated that the novel histone variant *HHT1* can partially fulfill *HHT2* and *HHT21*’s function | [(Rai et al., 2019)](https://paperpile.com/c/CEBw8c/zzRF5) |
| *C. albicans* | General Biology | *SSY1* | HDR-guided *NAT* cassette replacement of the gene | Cellular uptake of ornithine increased | [(Silao et al., 2019)](https://paperpile.com/c/CEBw8c/rGNi1) |
| *C. albicans* | General Biology | *CDC8* | HDR resulting in the 36 bp deletion of the Ca-loop | Doubling time increased | [(Huang et al., 2019a)](https://paperpile.com/c/CEBw8c/a46dk) |
| *C. albicans* | General Biology | *ASD* | HDR-guided *NAT* cassette replacement of the gene | Inability to create mutants indicates gene essentiality | [(Dahal et al., 2020)](https://paperpile.com/c/CEBw8c/uQibj) |
| *C. albicans* | General Biology | *HOF1* | HDR-guided *HIS1* cassette replacement of the gene | Sensitivity to MMS increased  Genome stability decreased | [(Feng et al., 2020a)](https://paperpile.com/c/CEBw8c/b6lF9) |
| *C. albicans* | General Biology | *DOT6* | HDR-guided repair that introduces a premature STOP codon | Growth on non-fermentable carbon sources reduced  *DBP7* and *KRE33* expression, decreased when grown on glycerol or lactate | [(Chaillot et al., 2019)](https://paperpile.com/c/CEBw8c/QKiU5) |
| *C. auris* | General Biology | *HSP90* | HDR-guided TetO cassette replacement of the promoter  This is to cause overexpression when not Tet is added or repression when Tet is added | Cell viability reduced when gene is repressed | [(Kim et al., 2019)](https://paperpile.com/c/CEBw8c/tvbMO) |
| *C. glabrata* | General Biology | *CDC43* | HDR-guided repair that introduces a premature STOP codon | Inability to create mutants indicate gene essentiality | [(Sun et al., 2020)](https://paperpile.com/c/CEBw8c/69R8G) |
| *C. glabrata* | General Biology | *MSH2* | HDR-guided repair inserted a *NAT*-*yGFP* cassette | Genome mutation rate increased as indicated by GFP expression | [(Shor et al., 2019)](https://paperpile.com/c/CEBw8c/aTy14) |
| *C. lusitaniae*    *C. glabrata*  *C. auris* | General Biology | *clCAT1*  *cgCAT1*  *caCAT1* | RNP-HDR-guided *SAT* cassette replacement of the gene | Sensitivity to hydrogen peroxide increased | [(Grahl et al., 2017)](https://paperpile.com/c/CEBw8c/XjxeL) |
| *C. parapsilosis* | General Biology | *DAL81* | HDR-guided repair that introduces a premature STOP codon | Growth rate unaffected  *ARG* genes upregulated | [(Turner et al., 2018)](https://paperpile.com/c/CEBw8c/Q85V7) |
| *C. albicans* | Immune System | *RAD23* | HDR-guided *HIS3* cassette replacement of the gene | Survival in macrophages reduced | [(Feng et al., 2020b)](https://paperpile.com/c/CEBw8c/cY81u) |
| *C. albicans* | Immune System | *MIG1/MIG2* | HDR-guided *NAT* cassette replacement of the gene | Virulence in macrophages decreased | [(Lagree et al., 2020)](https://paperpile.com/c/CEBw8c/SDkdo) |
| *C. albicans* | Immune System | *PUT1*  *PUT2*  *PUT3*  *PUT1/PUT2* | HDR-guided *NAT* cassette replacement of the gene | Virulence in macrophages decreased | [(Silao et al., 2019)](https://paperpile.com/c/CEBw8c/rGNi1) |
| *C. albicans* | Immune System | *CHO1* (as a single-gene deletion in wild type and secondary mutation in *sur7*Δ strain) | HDR-guided *SAT* cassette with transient CRISPR-Cas9 system | Copper-mediated killing of cells involves interactions of Cu with phosphatidylserine on plasma membrane | [(Douglas and Konopka, 2019)](https://paperpile.com/c/CEBw8c/6W0eP) |
| *C. albicans* | Immune System | *RAC1*, *MKC1* in both wild-type and *cho1*Δ/Δ strains, | HDR-guided *NAT* cassette replacement of the gene | ß-glucan unmasking occurs with upregulation of Cdc42-Cek1  pathway, not Rho1-Mkc1 pathway | [(Chen et al., 2019)](https://paperpile.com/c/CEBw8c/Ntc9R) |
| *C. albicans* | Immune System | *MNN4*-like gene family (single and octuple mutant) | HDR-guided *NAT* cassette replacement of the gene | Macrophage uptake of octuple mutant reduced | [(González-Hernández et al., 2017)](https://paperpile.com/c/CEBw8c/NiEe9) |
| *C. auris* | Immune System | *Als3* homologues*:*  *PIS50650.1*  *PIS50263.1*  *XP_018167572.2* | HDR-guided *SAT* cassette replacement of the gene using RNP | Pathogen recognition to adhesin-specific antibodies reduced | [(Singh et al., 2019)](https://paperpile.com/c/CEBw8c/ISUbc) |

[Chaillot, J., Tebbji, F., Mallick, J., and Sellam, A. (2019). Integration of Growth and Cell Size via the TOR Pathway and the Dot6 Transcription Factor in Candida albicans. *Genetics* 211, 637–650. doi:](http://paperpile.com/b/CEBw8c/QKiU5)[10.1534/genetics.118.301872](http://dx.doi.org/10.1534/genetics.118.301872)[.](http://paperpile.com/b/CEBw8c/QKiU5)

[Chen, T., Jackson, J. W., Tams, R. N., Davis, S. E., Sparer, T. E., and Reynolds, T. B. (2019). Exposure of Candida albicans β (1,3)-glucan is promoted by activation of the Cek1 pathway. *PLoS Genet.* 15, e1007892.](http://paperpile.com/b/CEBw8c/Ntc9R)

[Chen, Y., Mallick, J., Maqnas, A., Sun, Y., Choudhury, B. I., Côte, P., et al. (2018). Chemogenomic Profiling of the Fungal Pathogen Candida albicans. *Antimicrob. Agents Chemother.* 62. doi:](http://paperpile.com/b/CEBw8c/cwxQo)[10.1128/AAC.02365-17](http://dx.doi.org/10.1128/AAC.02365-17)[.](http://paperpile.com/b/CEBw8c/cwxQo)

[Dahal, G. P., Launder, D., McKeone, K. M. M., Hunter, J. P., Conti, H. R., and Viola, R. E. (2020). Aspartate semialdehyde dehydrogenase inhibition suppresses the growth of the pathogenic fungus Candida albicans. *Drug Dev. Res.* doi:](http://paperpile.com/b/CEBw8c/uQibj)[10.1002/ddr.21682](http://dx.doi.org/10.1002/ddr.21682)[.](http://paperpile.com/b/CEBw8c/uQibj)

[de San Vicente, K. M., Schröder, M. S., Lombardi, L., Iracane, E., and Butler, G. (2019). Correlating Genotype and Phenotype in the Asexual Yeast Implicates in Sensitivity to Caffeine. *G3*  9, 3035–3043.](http://paperpile.com/b/CEBw8c/1plUg)

[Douglas, L. M., and Konopka, J. B. (2019). Plasma membrane architecture protects Candida albicans from killing by copper. *PLoS Genet.* 15, e1007911.](http://paperpile.com/b/CEBw8c/6W0eP)

[Enkler, L., Richer, D., Marchand, A. L., Ferrandon, D., and Jossinet, F. (2016). Genome engineering in the yeast pathogen Candida glabrata using the CRISPR-Cas9 system. *Sci. Rep.* 6, 35766.](http://paperpile.com/b/CEBw8c/iZ4XV)

[Evans, B. A., Smith, O. L., Pickerill, E. S., York, M. K., Buenconsejo, K. J. P., Chambers, A. E., et al. (2018). Restriction digest screening facilitates efficient detection of site-directed mutations introduced by CRISPR in C. albicans UME6. *PeerJ* 6, e4920. doi:](http://paperpile.com/b/CEBw8c/CkGo)[10.7717/peerj.4920](http://dx.doi.org/10.7717/peerj.4920)[.](http://paperpile.com/b/CEBw8c/CkGo)

[Feng, J., Islam, A., Bean, B., Feng, J., Sparapani, S., Shrivastava, M., et al. (2020a). Hof1 plays a checkpoint-related role in MMS-induced DNA damage response in Candida albicans. *Molecular Biology of the Cell* 31, 348–359. doi:](http://paperpile.com/b/CEBw8c/b6lF9)[10.1091/mbc.e19-06-0316](http://dx.doi.org/10.1091/mbc.e19-06-0316)[.](http://paperpile.com/b/CEBw8c/b6lF9)

[Feng, J., Yao, S., Dong, Y., Hu, J., Whiteway, M., and Feng, J. (2020b). Nucleotide Excision Repair Protein Rad23 Regulates Cell Virulence Independent of Rad4 in Candida albicans. *mSphere* 5. doi:](http://paperpile.com/b/CEBw8c/cY81u)[10.1128/mSphere.00062-20](http://dx.doi.org/10.1128/mSphere.00062-20)[.](http://paperpile.com/b/CEBw8c/cY81u)

[González-Hernández, R. J., Jin, K., Hernández-Chávez, M. J., Díaz-Jiménez, D. F., Trujillo-Esquivel, E., Clavijo-Giraldo, D. M., et al. (2017). Phosphomannosylation and the Functional Analysis of the Extended Candida albicans MNN4-Like Gene Family. *Frontiers in Microbiology* 8. doi:](http://paperpile.com/b/CEBw8c/NiEe9)[10.3389/fmicb.2017.02156](http://dx.doi.org/10.3389/fmicb.2017.02156)[.](http://paperpile.com/b/CEBw8c/NiEe9)

[Grahl, N., Demers, E. G., Crocker, A. W., and Hogan, D. A. (2017). Use of RNA-Protein Complexes for Genome Editing in Non- Species. *mSphere* 2. doi:](http://paperpile.com/b/CEBw8c/XjxeL)[10.1128/mSphere.00218-17](http://dx.doi.org/10.1128/mSphere.00218-17)[.](http://paperpile.com/b/CEBw8c/XjxeL)

[Hollomon, J. M., Grahl, N., Willger, S. D., Koeppen, K., and Hogan, D. A. (2016). Global Role of Cyclic AMP Signaling in pH-Dependent Responses in. *mSphere* 1. doi:](http://paperpile.com/b/CEBw8c/vD3UV)[10.1128/mSphere.00283-16](http://dx.doi.org/10.1128/mSphere.00283-16)[.](http://paperpile.com/b/CEBw8c/vD3UV)

[Hou, X., Healey, K. R., Shor, E., Kordalewska, M., Ortigosa, C. J., Paderu, P., et al. (2019). Novel FKS1 and FKS2 modifications in a high-level echinocandin resistant clinical isolate of Candida glabrata. *Emerging Microbes & Infections* 8, 1619–1625. doi:](http://paperpile.com/b/CEBw8c/PBeEM)[10.1080/22221751.2019.1684209](http://dx.doi.org/10.1080/22221751.2019.1684209)[.](http://paperpile.com/b/CEBw8c/PBeEM)

[Huang, C.-Y., Chen, Y.-C., Wu-Hsieh, B. A., Fang, J.-M., and Chang, Z.-F. (2019a). The Ca-loop in thymidylate kinase is critical for growth and contributes to pyrimidine drug sensitivity of. *J. Biol. Chem.* 294, 10686–10697.](http://paperpile.com/b/CEBw8c/a46dk)

[Huang, M. Y., Woolford, C. A., May, G., Joel McManus, C., and Mitchell, A. P. (2019b). Circuit diversification in a biofilm regulatory network. *PLOS Pathogens* 15, e1007787. doi:](http://paperpile.com/b/CEBw8c/Ftksw)[10.1371/journal.ppat.1007787](http://dx.doi.org/10.1371/journal.ppat.1007787)[.](http://paperpile.com/b/CEBw8c/Ftksw)

[Kannan, A., Asner, S. A., Trachsel, E., Kelly, S., Parker, J., and Sanglard, D. (2019). Comparative Genomics for the Elucidation of Multidrug Resistance in Candida lusitaniae. *MBio* 10. doi:](http://paperpile.com/b/CEBw8c/6wvKE)[10.1128/mBio.02512-19](http://dx.doi.org/10.1128/mBio.02512-19)[.](http://paperpile.com/b/CEBw8c/6wvKE)

[Kapoor, M., Moloney, M., Soltow, Q. A., Pillar, C. M., and Shaw, K. J. (2019). Evaluation of Resistance Development to the Gwt1 Inhibitor Manogepix (APX001A) in Species. *Antimicrob. Agents Chemother.* 64. doi:](http://paperpile.com/b/CEBw8c/DYaC0)[10.1128/AAC.01387-19](http://dx.doi.org/10.1128/AAC.01387-19)[.](http://paperpile.com/b/CEBw8c/DYaC0)

[Kim, S. H., Iyer, K. R., Pardeshi, L., Muñoz, J. F., Robbins, N., Cuomo, C. A., et al. (2019). Genetic Analysis of Implicates Hsp90 in Morphogenesis and Azole Tolerance and Cdr1 in Azole Resistance. *MBio* 10. doi:](http://paperpile.com/b/CEBw8c/tvbMO)[10.1128/mBio.02529-18](http://dx.doi.org/10.1128/mBio.02529-18)[.](http://paperpile.com/b/CEBw8c/tvbMO)

[Lagree, K., Woolford, C. A., Huang, M. Y., May, G., McManus, C. J., Solis, N. V., et al. (2020). Roles of Candida albicans Mig1 and Mig2 in glucose repression, pathogenicity traits, and SNF1 essentiality. *PLoS Genet.* 16, e1008582.](http://paperpile.com/b/CEBw8c/SDkdo)

[Lee, K. K., Kubo, K., Abdelaziz, J. A., Cunningham, I., de Silva Dantas, A., Chen, X., et al. (2018). Yeast species-specific, differential inhibition of β-1,3-glucan synthesis by poacic acid and caspofungin. *Cell Surf* 3, 12–25.](http://paperpile.com/b/CEBw8c/Hgp4A)

[Liu, Z., and Myers, L. C. (2017a). Candida albicans Swi/Snf and Mediator Complexes Differentially Regulate Mrr1-Induced Expression and Fluconazole Resistance. *Antimicrob. Agents Chemother.* 61. doi:](http://paperpile.com/b/CEBw8c/tBaI4)[10.1128/AAC.01344-17](http://dx.doi.org/10.1128/AAC.01344-17)[.](http://paperpile.com/b/CEBw8c/tBaI4)

[Liu, Z., and Myers, L. C. (2017b). Mediator Tail Module Is Required for Tac1-Activated Expression and Azole Resistance in Candida albicans. *Antimicrob. Agents Chemother.* 61. doi:](http://paperpile.com/b/CEBw8c/kZEWx)[10.1128/AAC.01342-17](http://dx.doi.org/10.1128/AAC.01342-17)[.](http://paperpile.com/b/CEBw8c/kZEWx)

[Lu, Y., Su, C., Ray, S., Yuan, Y., and Liu, H. (2019). CO Signaling through the Ptc2-Ssn3 Axis Governs Sustained Hyphal Development of Candida albicans by Reducing Ume6 Phosphorylation and Degradation. *MBio* 10. doi:](http://paperpile.com/b/CEBw8c/KOkyz)[10.1128/mBio.02320-18](http://dx.doi.org/10.1128/mBio.02320-18)[.](http://paperpile.com/b/CEBw8c/KOkyz)

[Mendelsohn, S., Pinsky, M., Weissman, Z., and Kornitzer, D. (2017). Regulation of the Hypha-Inducing Transcription Factor Ume6 by the CDK1 Cyclins Cln3 and Hgc1. *mSphere* 2. doi:](http://paperpile.com/b/CEBw8c/uGOew)[10.1128/mSphere.00248-16](http://dx.doi.org/10.1128/mSphere.00248-16)[.](http://paperpile.com/b/CEBw8c/uGOew)

[Min, K., Biermann, A., Hogan, D. A., and Konopka, J. B. (2018). Genetic Analysis of Family Transcription Factors in Using New CRISPR-Cas9 Approaches. *mSphere* 3. doi:](http://paperpile.com/b/CEBw8c/FSuug)[10.1128/mSphere.00545-18](http://dx.doi.org/10.1128/mSphere.00545-18)[.](http://paperpile.com/b/CEBw8c/FSuug)

[Morio, F., Lombardi, L., Binder, U., Loge, C., Robert, E., Graessle, D., et al. (2019). Precise genome editing using a CRISPR-Cas9 method highlights the role of CoERG11 amino acid substitutions in azole resistance in Candida orthopsilosis. *J. Antimicrob. Chemother.* 74, 2230–2238.](http://paperpile.com/b/CEBw8c/Cf31l)

[Naseem, S., Min, K., Spitzer, D., Gardin, J., and Konopka, J. B. (2017). Regulation of Hyphal Growth and N-Acetylglucosamine Catabolism by Two Transcription Factors in Candida albicans. *Genetics* 206, 299–314. doi:](http://paperpile.com/b/CEBw8c/VUTou)[10.1534/genetics.117.201491](http://dx.doi.org/10.1534/genetics.117.201491)[.](http://paperpile.com/b/CEBw8c/VUTou)

[Nishimoto, A. T., Zhang, Q., Hazlett, B., Morschhäuser, J., and Rogers, P. D. (2019). Contribution of Clinically Derived Mutations in the Gene Encoding the Zinc Cluster Transcription Factor Mrr2 to Fluconazole Antifungal Resistance and Expression in. *Antimicrob. Agents Chemother.* 63. doi:](http://paperpile.com/b/CEBw8c/1FAUz)[10.1128/AAC.00078-19](http://dx.doi.org/10.1128/AAC.00078-19)[.](http://paperpile.com/b/CEBw8c/1FAUz)

[Rai, L. S., Singha, R., Sanchez, H., Chakraborty, T., Chand, B., Bachellier-Bassi, S., et al. (2019). The Candida albicans biofilm gene circuit modulated at the chromatin level by a recent molecular histone innovation. *PLoS Biol.* 17, e3000422.](http://paperpile.com/b/CEBw8c/zzRF5)

[Rybak, J. M., Doorley, L. A., Nishimoto, A. T., Barker, K. S., Palmer, G. E., and David Rogers, P. (2019). Abrogation of Triazole Resistance upon Deletion of CDR1 in a Clinical Isolate of Candida auris. *Antimicrobial Agents and Chemotherapy* 63. doi:](http://paperpile.com/b/CEBw8c/EEWfV)[10.1128/aac.00057-19](http://dx.doi.org/10.1128/aac.00057-19)[.](http://paperpile.com/b/CEBw8c/EEWfV)

[Rybak, J. M., Muñoz, J. F., Barker, K. S., Parker, J. E., Esquivel, B. D., Berkow, E. L., et al. (2020). Mutations in : a Novel Genetic Determinant of Clinical Fluconazole Resistance in Candida auris. *MBio* 11. doi:](http://paperpile.com/b/CEBw8c/yjoBR)[10.1128/mBio.00365-20](http://dx.doi.org/10.1128/mBio.00365-20)[.](http://paperpile.com/b/CEBw8c/yjoBR)

[Shapiro, R. S., Chavez, A., Porter, C. B. M., Hamblin, M., Kaas, C. S., DiCarlo, J. E., et al. (2018). A CRISPR–Cas9-based gene drive platform for genetic interaction analysis in Candida albicans. *Nature Microbiology* 3, 73–82. doi:](http://paperpile.com/b/CEBw8c/800lv)[10.1038/s41564-017-0043-0](http://dx.doi.org/10.1038/s41564-017-0043-0)[.](http://paperpile.com/b/CEBw8c/800lv)

[Shor, E., Schuyler, J., and Perlin, D. S. (2019). A Novel, Drug Resistance-Independent, Fluorescence-Based Approach To Measure Mutation Rates in Microbial Pathogens. *MBio* 10. doi:](http://paperpile.com/b/CEBw8c/aTy14)[10.1128/mBio.00120-19](http://dx.doi.org/10.1128/mBio.00120-19)[.](http://paperpile.com/b/CEBw8c/aTy14)

[Silao, F. G. S., Ward, M., Ryman, K., Wallström, A., Brindefalk, B., Udekwu, K., et al. (2019). Mitochondrial proline catabolism activates Ras1/cAMP/PKA-induced filamentation in Candida albicans. *PLoS Genet.* 15, e1007976.](http://paperpile.com/b/CEBw8c/rGNi1)

[Singh, S., Uppuluri, P., Mamouei, Z., Alqarihi, A., Elhassan, H., French, S., et al. (2019). The NDV-3A vaccine protects mice from multidrug resistant Candida auris infection. *PLoS Pathog.* 15, e1007460.](http://paperpile.com/b/CEBw8c/ISUbc)

[Sun, Q., Xiong, K., Yuan, Y., Yu, J., Yang, L., Shen, C., et al. (2020). Inhibiting Fungal Echinocandin Resistance by Small-Molecule Disruption of Geranylgeranyltransferase Type I Activity. *Antimicrob. Agents Chemother.* 64. doi:](http://paperpile.com/b/CEBw8c/69R8G)[10.1128/AAC.02046-19](http://dx.doi.org/10.1128/AAC.02046-19)[.](http://paperpile.com/b/CEBw8c/69R8G)

[Turner, S. A., Ma, Q., Ola, M., Martinez de San Vicente, K., and Butler, G. (2018). Dal81 Regulates Expression of Arginine Metabolism Genes in. *mSphere* 3. doi:](http://paperpile.com/b/CEBw8c/Q85V7)[10.1128/mSphere.00028-18](http://dx.doi.org/10.1128/mSphere.00028-18)[.](http://paperpile.com/b/CEBw8c/Q85V7)

[Veri, A. O., Miao, Z., Shapiro, R. S., Tebbji, F., O’Meara, T. R., Kim, S. H., et al. (2018). Tuning Hsf1 levels drives distinct fungal morphogenetic programs with depletion impairing Hsp90 function and overexpression expanding the target space. *PLoS Genet.* 14, e1007270.](http://paperpile.com/b/CEBw8c/sZCqc)

[Vyas, V. K., Inmaculada Barrasa, M., and Fink, G. R. (2015). A Candida albicans CRISPR system permits genetic engineering of essential genes and gene families. *Science Advances* 1, e1500248. doi:](http://paperpile.com/b/CEBw8c/pfPm)[10.1126/sciadv.1500248](http://dx.doi.org/10.1126/sciadv.1500248)[.](http://paperpile.com/b/CEBw8c/pfPm)

[Wijnants, S., Riedelberger, M., Penninger, P., Kuchler, K., and Van Dijck, P. (2020). Sugar Phosphorylation Controls Carbon Source Utilization and Virulence of Candida albicans. *Frontiers in Microbiology* 11. doi:](http://paperpile.com/b/CEBw8c/D3ij7)[10.3389/fmicb.2020.01274](http://dx.doi.org/10.3389/fmicb.2020.01274)[.](http://paperpile.com/b/CEBw8c/D3ij7)

[Williams, R. B., and Lorenz, M. C. (2020). Multiple Alternative Carbon Pathways Combine To Promote Candida albicans Stress Resistance, Immune Interactions, and Virulence. *MBio* 11. doi:](http://paperpile.com/b/CEBw8c/hixrN)[10.1128/mBio.03070-19](http://dx.doi.org/10.1128/mBio.03070-19)[.](http://paperpile.com/b/CEBw8c/hixrN)

[Woolford, C. A., Lagree, K., Xu, W., Aleynikov, T., Adhikari, H., Sanchez, H., et al. (2016). Bypass of Candida albicans Filamentation/Biofilm Regulators through Diminished Expression of Protein Kinase Cak1. *PLoS Genet.* 12, e1006487.](http://paperpile.com/b/CEBw8c/Oi42K)

[Zoppo, M., Di Luca, M., Franco, M., Rizzato, C., Lupetti, A., Stringaro, A., et al. (2020). CpALS4770 and CpALS4780 contribution to the virulence of Candida parapsilosis. *Microbiol. Res.* 231, 126351.](http://paperpile.com/b/CEBw8c/rYBkW)

[Zoppo, M., Di Luca, M., Villarreal, S. N., Poma, N., Inmaculada Barrasa, M., Bottai, D., et al. (2019). A CRISPR/Cas9-based strategy to simultaneously inactivate the entire ALS gene family in Candida orthopsilosis. *Future Microbiology* 14, 1383–1396. doi:](http://paperpile.com/b/CEBw8c/R9XLS)[10.2217/fmb-2019-0168](http://dx.doi.org/10.2217/fmb-2019-0168)[.](http://paperpile.com/b/CEBw8c/R9XLS)

[Zoppo, M., Lombardi, L., Rizzato, C., Lupetti, A., Bottai, D., Papp, C., et al. (2018). CORT0C04210 is required for Candida orthopsilosis adhesion to human buccal cells. *Fungal Genet. Biol.* 120, 19–29.](http://paperpile.com/b/CEBw8c/HKQir)
